# Global characterization of biosynthetic gene clusters in non-model eukaryotes using domain architectures

**DOI:** 10.1038/s41598-023-50095-3

**Published:** 2024-01-17

**Authors:** Taehyung Kwon, Blake T. Hovde

**Affiliations:** Genomics and Bioanalytics Group, Bioscience Division, Los Alamos National Laboratory, Los Alamos, NM USA

**Keywords:** Data mining, Pharmacogenomics, Comparative genomics

## Abstract

The majority of pharmaceuticals are derived from natural products, bioactive compounds naturally synthesized by organisms to provide evolutionary advantages. Although the rich evolutionary history of eukaryotic algal species implicates a high potential for natural product-based drug discovery, it remains largely untouched. This study investigates 2762 putative biosynthetic gene clusters (BGCs) from 212 eukaryotic algal genomes. To analyze a vast set of structurally diverse BGCs, we employed comparative analysis based on the vectorization of biosynthetic domains, referred to as biosynthetic domain architecture (BDA). By characterizing core biosynthetic machineries through BDA, we identified key BDAs of modular BGCs in diverse eukaryotes and introduced 16 candidate modular BGCs with similar BDAs to previously validated BGCs. This study provides a global characterization of eukaryotic algal BGCs, offering an alternative to laborious manual curation for BGC prioritization.

## Introduction

Natural products, also known as secondary metabolites, are molecules naturally synthesized by a wide range of organisms to confer evolutionary advantages to the host^1^. These molecules undergo optimization within the host system to enhance their bioactivity and reduce toxicity^1^. Importantly, natural products constitute a vital source for drug discovery, accounting for over 60% of drugs approved by the US Food and Drug Administration (FDA) between 1981 and 2019^2^. The functional diversity of natural products enables them to offer a broad range of benefits to public health^1,3^. However, this functional diversity often comes at the cost of intricate molecular structures^4^, thereby complicating the drug discovery process.

Traditional natural product-based drug discovery has heavily relied on accurate yet costly biochemical characterization. Recent studies suggest that genetic potential for expression of natural products in an organism may exceed the information provided by biochemical snapshots of the organism, particularly considering the conditional nature of natural product expression^5,6^. At the DNA level, the focus of natural product discovery has shifted towards finding a set of adjacent genes that encode biosynthetic enzymes, known as biosynthetic gene clusters (BGCs)^6^. With the decreasing costs of genome sequencing, computational detection of BGCs has become an crucial step in natural product discovery^7,8^. This computational approach utilizes knowledge accumulated from previously identified BGCs^9,10^. Today, the intersection of bioinformatic advancements and a deepened understanding of BGCs has given rise to multiple computational tools for the detection of BGCs^8^.

Eukaryotic algae, representing a polyphyletic group of diverse photosynthetic organisms, have obtained recognition as potential manufacturers of a wide array of natural products^11,12^. Given their rich evolutionary histories and adaptation to unique ecological niches, these eukaryotes may have developed natural products as part of their interactions with other microorganisms, serving functions such as chemical defense against predators^12^. Eukaryotic genomes are capable of encoding natural products of high molecular masses, but their complex genomic structure can work as a barrier in BGC detection^13^. However, genomic resources of the majority of non-model eukaryotes remain limited in publicly available databases^14^. Consequently, eukaryotic BGCs, excluding fungal BGCs, are reported to be scarce in prominent BGC databases like the Minimum Information about a Biosynthetic Gene cluster (MIBiG)^14,15^. The most recent update from the MIBiG database reveals an enrichment of bacterial (n = 2011, 80.38%) and fungal BGCs (n = 441, 17.63%) among their 2502 BGCs, with a relatively low representation of other eukaryotic BGCs^15^.

Homologous genes often exhibit low sequence identities across a broad phylogenetic spectrum. The profile Hidden Markov Model (pHMM) employs probabilistic models that facilitates protein homology search despite low sequence identities, known to be particularly useful for protein domains^16,17^. For this reason, the pHMM-based homology search has been integrated into cutting-edge BGC detection tools such as PRISM^18^ and antiSMASH^16^. Notably, antiSMASH stands out as one of the most robust detection tools in the field, equipped with an extensive set of pHMMs for biosynthetic domains^16^. The growing database of antiSMASH pHMMs enables highly inclusive BGC detection across a wide range of organisms^16^, endorsed by the MIBiG database^15^. Although the development of computational BGC detection has expedited natural product discovery, these tools often yield a substantial number of structurally diverse putative BGCs. This, coupled with a increasing stream of published genome sequences^6^, necessitates a strategy to effectively compare and prioritize a massive number of computationally detected BGCs^19^. In this regard, BGC prioritization becomes a valuable option to reduce the cost and time associated with bench work in natural product discovery^5^. However, there have been only a handful of published attempts to prioritize promising BGCs, and these efforts have focused on proximate genes that are not directly involved in natural product biosynthesis^5,20,21^.

The inherent structural diversity of putative BGCs offers researchers a diversified catalog of natural products for drug discovery. However, this structural diversity poses a challenge by impeding comparative analysis of BGCs, which leads to a bottleneck in realization of natural products^5,22^. To address this challenge in large-scale comparative analysis of BGCs, BiG-SCAPE and BiG-SLiCE have successfully adopted vectorization of biosynthetic domains using bacterial BGCs^21,23^. These programs extract frequencies of biosynthetic domains from the vectorized biosynthetic domains obtained from antiSMASH^21,23^. In addition, BiG-SCAPE implemented the co-occurrence of 2-mers of biosynthetic domains^21^. While these metrics are optimized for comparative analysis and clustering of a large number of BGCs, they are limited to partial snapshot of conservations of biosynthetic machineries, particularly for modular BGCs composed of more than ten biosynthetic domains such as polyketide synthases (PKS) and non-ribosomal peptide synthetases (NRPS)^19^.

In this study, we characterized diverse BGCs from 212 eukaryotic algal genome sequences with the assistance of experimentally characterized MIBiG BGCs. To address phylogenetic divergences within the broad spectrum of eukaryotes, we employed vectorized biosynthetic domains, hereafter referred to as the biosynthetic domain architecture (BDA)^24–26^, to investigate the conservation of biosynthetic machineries. We performed pair-wise alignment of BDAs with a scoring matrix of biosynthetic domain similarities, which mitigates the challenges posed by variable sequence identities among BGCs found across phylogenetically distinct organisms. Furthermore, we adopted an alternative BGC prioritization by combining this comparative analysis with the experimentally characterized MIBiG BGCs. As a result, we identified the key BDAs of eukaryotic algal BGCs, as well as promising eukaryotic algal BGCs that share highly conserved BDAs with the MIBiG BGCs. This study enables a global characterization of key biosynthetic machineries in modular BGCs across a wide range of eukaryotes and facilitates the prioritization of promising modular BGCs based on the conservation of biosynthetic machineries.

## Results

### Genome sequences and annotation

**Figure 1:**
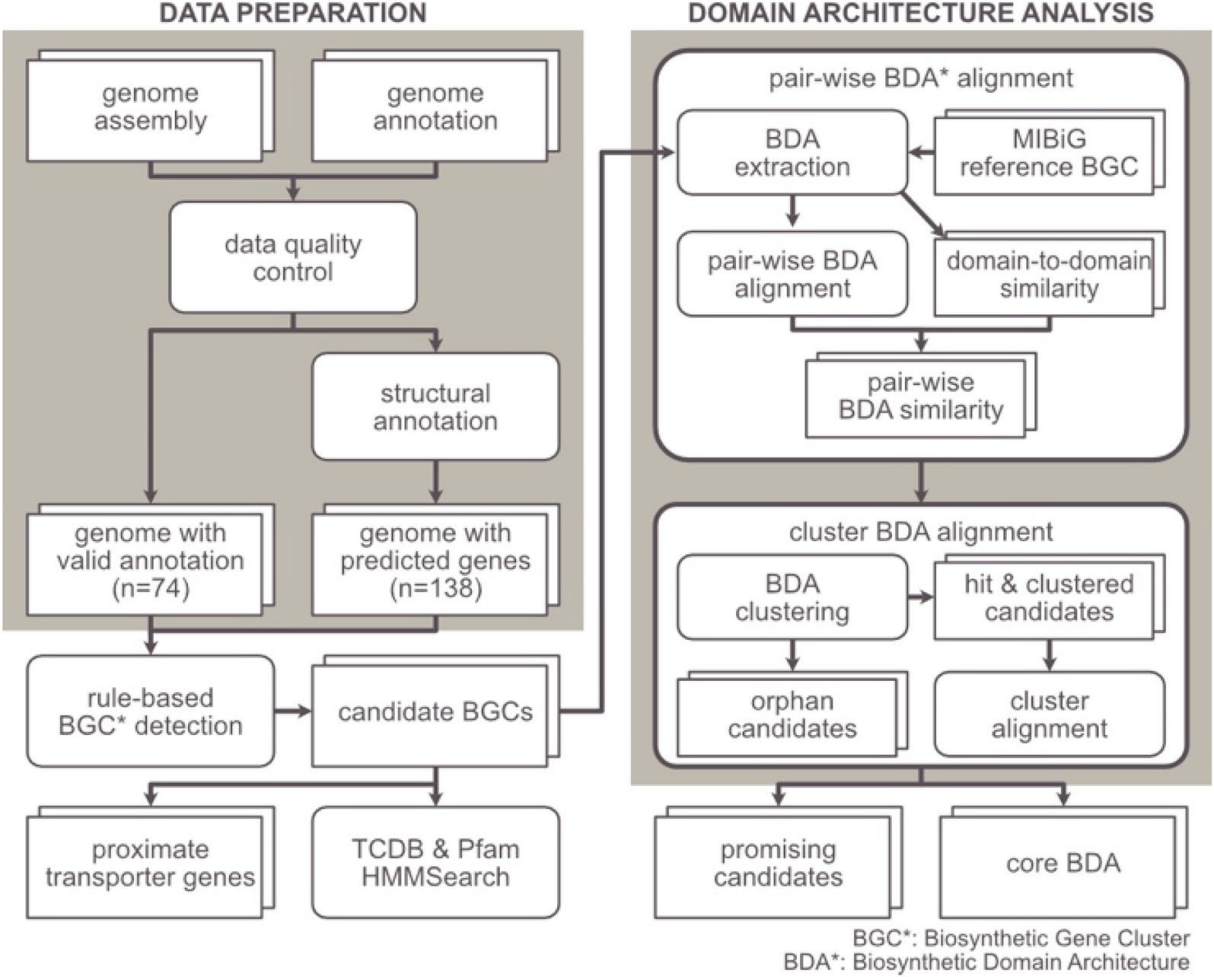
Flowchart of the study.

The data preparation of the eukaryotic algal genome database used in this study is outlined in Kwon et al.^14^. Initially, we retrieved 257 publicly available genome sequences and annotations of eukaryotic algal species from public databases, then filtered them out or compensated for low-quality or missing information prior to the main analyses (Fig. 1). We first excluded 29 assemblies from less reliable sources such as complex metagenomic samples. Next, we excluded five genome assemblies with contig N50 values smaller than 1.608 kb, the median gene length of the eukaryotic algae calculated from genome annotations of 17 genome assemblies with contig N50 values of 100 kb or more^14^. We also excluded genome assemblies with BUSCO missing rates over 75%^14^. As a result, we selected 212 eukaryotic algal genome assemblies (Table S1) to be used in the genome mining.

Among 212 genomes, 83 possessed the official structural annotations from the original authors (Table S1). We first excluded five annotations with (i) anomalies in core annotation features, and/or (ii) discordances between genome assembly, genome annotation, and protein sequences (denoted as “anomaly” in the *Annotation* column of Table S1). We additionally excluded one more annotation based on its high missing rate over 50% in BUSCO protein mode analysis with automatically selected lineages (denoted as “busco quality fail” in the *Annotation* column of Table S1). Lastly, we examined the validity of annotations for antiSMASH analysis^16^. We selected the longest isoform of each transcript in genome annotations, then we input these genome annotations into the standard antiSMASH analysis pipeline. This results in the removal of three annotations that failed validation (denoted as “validity check fail” in the *Annotation* column of Table S1). As a result, we used the official annotations for 74 genome assemblies (denoted as “official annotation” in the *Annotation* column of Table S1). Otherwise, we generated predicted gene sets for 138 genome assemblies without annotations, using Braker2^27^ and OrthoDB v10^28^ as described in Kwon et al.^14^.

### Establishment of the experimentally characterized biosynthetic gene cluster set

**Figure 2:**
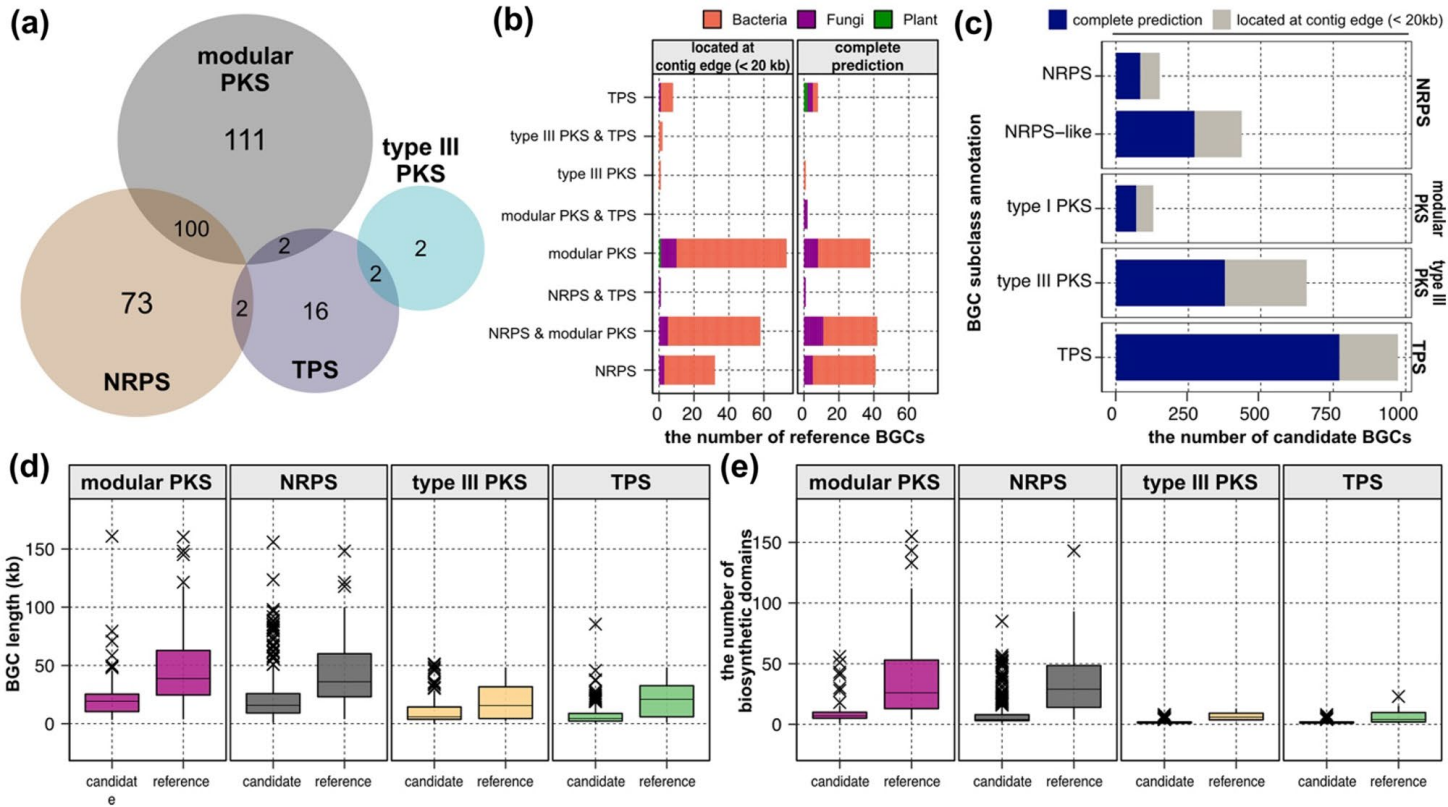
Summary of candidate and reference biosynthetic gene clusters. (**a**) A Venn diagram of classes of the reference BGCs. (**b**) The number of reference BGCs by class. Each color indicates taxonomic classification of source organism. Left panel displays BGCs located at contig edge regions. (**c**) The number of the candidate BGCs by classes. Grey colored bar displays BGCs located at contig edge regions. (**d**,**e**) Class-wise distributions of (**d**) BGC lengths and (**e**) the number of biosynthetic domains.

For the comparative analyses across putative BGCs found in this study, we retrieved a total of 427 experimentally characterized BGCs from 2502 MIBiG 3.1 BGC entries^15^. These 427 MIBiG BGCs were annotated as “complete” and supported by at least one piece of experimental evidence in MIBiG JSON metadata. We determined classes and subclasses of these experimentally characterized BGCs based on the MIBiG JSON metadata. We classified the experimentally characterized BGCs into four classes, narrowing to a total of 175 NRPSs, 213 modular PKSs, 4 type III PKSs, 22 Terpene Synthases (TPS) (Fig. 2a) and 119 others. We note that each class annotation of an experimentally characterized BGC can comprise multiple compound properties (Fig. 2a). For downstream analyses, we only included 308 experimentally characterized BGCs belonging to four major classes (NRPS, modular PKS, type III PKS, and TPS), hereafter referred to as “reference BGCs” (Table S2).

The reference BGCs were supported by at least one of the following experimental procedures (see the *evidence* column of Table S2): knock-out studies, enzymatic assays, heterologous expression, and gene expression correlated with compound production^15^. Among 308 reference BGCs, the majority (n = 256) originated from various bacterial species, and 51 reference BGCs originated from eukaryotes; 49 from fungal species and two from *Arabidopsis thaliana* (Table S2 and Fig. 2b). For one reference BGC (BGC0001875), the source organism information remained unidentified (Table S2). A total of 47 chemical properties of the compounds were currently annotated (see the *chemical activity* column of Table S2), and top five most frequent chemical activities in the reference BGCs were antibacterial, cytotoxic, antifungal, inhibitor, and surfactant (Fig. S1).

### Computational detection of eukaryotic algal biosynthetic gene clusters

We performed rule-based detection of BGCs using antiSMASH v6.1.1^16^, which finds a set of domains that are known to be specific to a certain BGC class. From 212 eukaryotic algal genomes, we detected a total of 2762 candidate BGCs (Fig. 1). To minimize fragmented BGC detection, we filtered out 286 BGCs detected from contigs/scaffolds shorter than 10 kb, leaving 2476 candidate BGCs (Table S3). BGCs from four genomes were completely excluded during this filtering step: *Pyropia_yezoensis*__U51, *Messastrum_gracile*__SEMC4, *Helicosporidium*_sp.__ATCC50920, and *Prototheca_bovis*__SAG2021. For the class-wise comparison with the reference BGCs, we classified the candidate BGCs into five BGC classes frequently found in the eukaryotic algal BGC dataset, using BGC subclass labels: two modular BGC classes (NRPS, n = 585; modular PKS, n = 129), two non-modular BGC classes (type III PKS, n = 658; terpene synthase or TPS, n = 973), and other BGCs (n = 131) (Fig. 2c and Table S3). NRPS candidates were composed NRPS and NRPS-like subclasses. Modular PKS candidates were only composed of type I PKS subclass, as type II PKS candidates were not detected. Other BGC candidates were excluded from the downstream analyses due to small numbers of BGC subclasses (Table S3).

The genome size exhibited a significant correlation with the number of the non-modular candidate BGCs (Spearman’s correlation coefficient *ρ* = 0.42, *p*-value < 2.2e^−16^ for type III PKS; *ρ* = 0.18, *p*-value = 0.0094 for TPS). However, there was no significant correlation between the genome size and the number of the modular candidate BGCs (NRPS and modular PKS) (Fig. S2). The contig N50 value was only significantly correlated with the number of the candidate TPSs (*ρ* = 0.33, *p*-value < 2.2e^−16^), but not with other BGCs (Fig. S3). In most BGC classes, genome size and genome continuity did not significantly affect the number of BGCs detected in the genome assembly. The continuity of genome assembly or the size of genome does not affect the number of detected modular BGCs.

To infer complete detection of each BGCs, we examined the location of BGCs within contigs; a BGC located at the contig edge that refers to 20 kb regions at 5′ or 3′ ends of contigs could be an incomplete detection. 39.3% of NRPS candidates (n = 230), 45.7% of modular PKS candidates (n = 59), 42.9% of type III PKS candidates (n = 282), and 20.8% of TPS candidates (n = 202) were located at contig edge (Fig. 2c). However, a large portion of the reference BGCs (56.92%, n = 175) were also located at the contig edge, and 164 of them were NRPSs and/or modular PKSs (Fig. 2b). This result indicates potential incomplete annotation in BGC detection, particularly in modular BGCs that often span long genomic regions.

We observed significant differences in BGC lengths between the candidates and references in NRPS (Wilcoxon rank-sum test *p*-value = 2.01e^−30^), modular PKS (*p*-value = 1.79e^−20^), and TPS (*p*-value = 7.31e^−6^), except for type III PKS (*p*-value = 0.40) (Fig. 2d). Similarly, we observed significant differences in the number of biosynthetic domains between candidates and references in NRPS (*p*-value = 5.68e^−59^), modular PKS (*p*-value = 3.42e^−35^), type III PKS (*p*-value = 1.06e^−3^), and TPS (*p*-value = 9.47e^−13^) (Fig. 2e). Lower *p*-values in modular BGCs suggest that modular BGCs contain higher variation between the candidate and reference sets. In addition, a large difference in the number of biosynthetic domains between candidate outliers and reference outliers (Fig. 2e) was not observed in the BGC lengths (Fig. 2d), which supports the structural difference of BGCs between the candidates and references.

### Canonical biosynthetic domains of biosynthetic gene clusters

**Figure 3:**
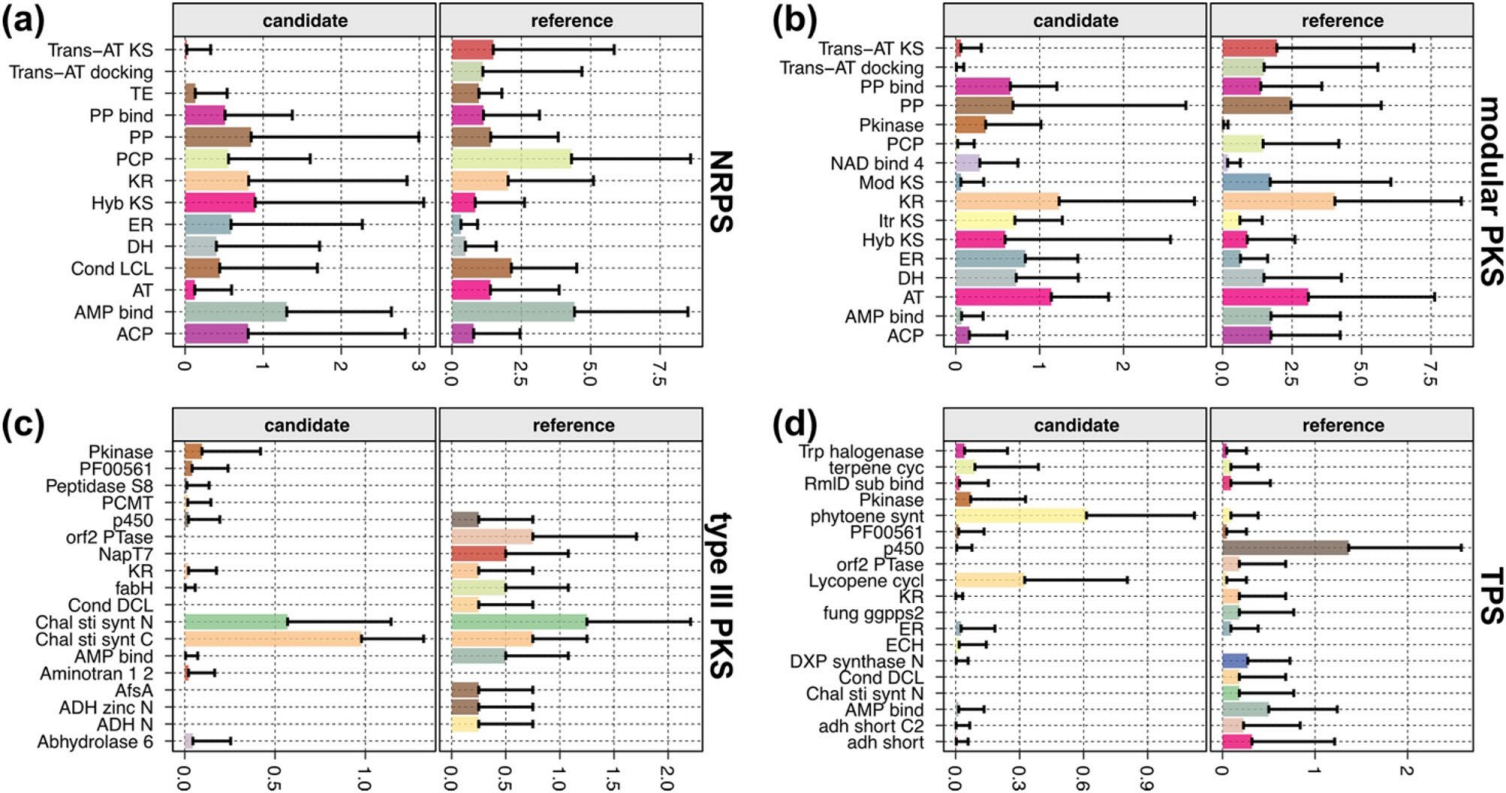
Biosynthetic domain composition of each BGC class. Domain frequencies of the ten most frequently observed biosynthetic domains in each reference category for (**a**) NRPS, (**b**) modular PKS, (**c**) type III PKS, and (**d**) TPS. Domain symbols are summarized in Table S4.

Combinations of diverse biosynthetic domains determine the biosynthetic mechanisms of natural products, acting as core functional units of BGCs^16^. To grasp the landscape of canonical biosynthetic domains of eukaryotic algal BGCs, we investigated biosynthetic domain compositions estimated by mean counts of biosynthetic domains, or referred to as domain frequencies. Domain frequency of *N* indicates that the domain appears *N* times on average within each BGC class. For each BGC class, top ten canonical domains for the reference BGCs and the candidate BGCs were summarized respectively (Fig. 3). Biosynthetic domain symbols are summarized in Table S4. Canonical domains of modular BGC classes are summarized in Text S1.

#### NRPS

The candidate and reference NRPSs were enriched with three NRPS domains: adenylation domain “AMP bind”, peptidyl-carrier protein “PCP”, and condensation domain “Cond LCL” (Fig. 3a). Roles of these domains in NRP biosynthesis are described in Text S1. Domain frequencies of these canonical NRPS domains were higher in the reference NRPSs (domain frequency of adenylation domain = 4.42; domain frequency of PCP = 4.31; domain frequency of condensation ^L^C_L_ = 2.14) (Fig. 3a), suggesting that the reference NRPSs are likely composed of multiple modules. In contrast, domain frequencies of PCP and condensation domains in the candidate NRPSs were lower than 1 (domain frequency of PCP = 0.56; domain frequency of condensation ^L^C_L_ = 0.44) (Fig. 3a). A large variance of the number of biosynthetic domains and lower domain frequencies in the candidate NRPSs (Fig. 3a) may have resulted from incomplete modules due to low continuity of eukaryotic algal genome assemblies. Although the non-modular NRPSs were often reported in bacterial NRPSs, we should also note that the candidate NRPSs may include the non-modular NRPSs^29^.

PCP was predominantly found in the reference NRPSs (domain frequency = 4.31). Instead, acyl carrier proteins that are often found in PKSs (“ACP” or phosphopantetheine acyl-carrier proteins “PP” and “PP bind”) were observed in similar or higher frequencies to the PCP (domain frequency = 0.56) in the candidate NRPSs (Fig. 3a): domain frequency of ACP = 0.81; domain frequency of PP = 0.85; domain frequency of PP bind = 0.51. Likewise, typical PKS biosynthetic domains were frequently found in the candidates: acyltransferase “AT” (domain frequency = 0.12), ketoreductase “KR” (0.82), hybrid ketosynthase (KS) “Hyb KS” (0.90), enoylreductase “ER” (0.59), and dehydratase “DH” (0.40) (Fig. 3a). Hybrid KS domains have been uniquely found in the NRPS-PKS hybrid modules that are composed of PKS domains to extend products from upstream NRPS modules^30^.

Although *trans*-AT KS domain is mainly found in PKS modules that operate with free-standing AT domains outside of the modules, *trans*-AT PKS modules are also known to form hybrids with NRPS modules^31,32^. While *trans*-acyltransferase KS domain (*trans*-AT KS) and *trans*-acyltransferase docking domain “Trans-AT docking” were rarely found in the candidate NRPSs (domain frequency of *trans*-AT KS = 0.022; domain frequency of *trans*-AT docking = 0), these domains were abundant in the reference NRPSs (domain frequency of *trans*-AT KS = 1.49; domain frequency of *trans*-AT docking = 1.11) (Fig. 3a). A large number of NRPS-PKS hybrids among the reference NRPSs (n = 100, Fig. 2a) and the abundance of *trans*-AT KSs in the reference NRPSs indicates the presence of *trans*-AT PKS-NRPS hybrids, which is scarce among the candidate NRPSs (Fig. 3a).

#### Modular PKS

PKSs operate and are structured in a modular manner with a set of canonical domains, similar to NRPSs. The candidate modular PKSs were detected based on the presence of two of canonical PKS domains: acyltransferase domain followed by one of various KS domains. Accordingly, these canonical domains were abundant in both candidate and reference modular PKSs (Fig. 3b). Various KS domains were found: hybrid KS, iterative KS “Itr KS”, modular KS “Mod KS”, and *trans*-AT KS (Fig. 3b). Roles of these domains in NRP biosynthesis are described in Text S1. In addition to the canonical domains, other tailoring PKS domains were abundant in both candidate and reference modular PKSs: ketoreductase, dehydratase, and enoylreductase. These domains can modify the acyl-carrier protein-bound substrates before being passed over to the next module^33^.

Other than hybrid KS being abundant in both candidate (domain frequency = 0.59) and reference modular PKSs (domain frequency = 0.89), the compositions of KS domains were different between the reference and candidate modular PKSs (Fig. 3b). While the candidate modular PKSs were enriched with iterative KS (domain frequency = 0.71), the reference modular PKSs were enriched with modular KS (domain frequency = 1.71) and *trans*-AT KS (domain frequency = 1.95) (Fig. 3b). Briefly, iterative KS domain works with a single iterative PKS module (Text S1). Although all subclasses of modular PKSs are known to be found across eukaryotes^33^, iterative KS domains are known to be predominantly found in the eukaryotic PKSs. This report concurs with the status of the MIBiG reference PKSs, as all of eukaryotic reference modular PKSs (n = 35) contained at least one iterative KS, whereas only 32.57% of the bacterial reference modular PKSs (n = 57) contained iterative KS (Table S2). Similarly, 87 among 129 candidate modular PKSs contained iterative KS (Table S3). This result supports that modular PKSs in fungal species as well as eukaryotic algal species share similar machineries.

#### Type III PKS

Different from modular manners observed in modular PKSs (type I or type II PKS), type III PKSs are operated by self-contained enzymes that accounts for starter, elongation, and cyclization of the substrate^34^. For example, Chalcone synthases and stilbene synthases are well-studied enzymes that are homologous to each other, in the sense of catalyzing tetraketide formation in plant polyketide synthesis from starter units^35^. In the rule-based detection process of antiSMASH, the candidate type III PKSs were detected based on the presence of chalcone/stilbene synthases “Chal sti synt”. Domain compositions of both reference and candidate type III PKSs were centered around N- and C-terminal dimers of chalcone/stilbene synthases (Fig. 3c and Fig. S4), different from those of modular BGCs (Fig. 3a,b). In addition to the canonical chalcone/stilbene synthases, type III PKSs modify the substrates with various tailoring enzymes^34^. We should note that one of four reference type III PKSs, BGC0000189, does not include canonical type III PKS enzymes (Table S2), suggesting erroneous MIBiG annotation as type III PKS. The original report of BGC0000189 identified presence of non-acetate starter unit ketosynthase III within the cluster, which predicts the BGC as a type II PKS^36^.

#### TPS

Similar to type III PKSs, TPSs are non-modular BGCs that are mainly composed of self-contained canonical enzymes that are known to synthesize terpenoids^37^. Accordingly, TPSs were enriched with these canonical domains, accompanied by various tailoring enzymes (Fig. 3d). The majority of candidate TPSs were structured based on phytoene synthase “phytoene synt” (domain frequency = 0.61) and lycopene cyclase “Lycopene cycl” (domain frequency = 0.32) that synthesize precursors of 40-carbon terpenes called carotenoids (Fig. 3d and Fig. S5)^38^. In contrast, these two TPS enzymes were rare in the reference TPSs (domain frequency of phytoene synthase = 0.091; domain frequency of lycopene cyclase = 0.045), while other canonical cyclization enzymes such as fungal geranylgeranyl diphosphate “fungi_ggpps2” (domain frequency = 0.18) were also found. Although terpene biosynthesis includes formation of multiple five-carbon hydrocarbon skeletons by a single canonical enzyme^39,40^, the backbones can be extensively modified by tailoring enzymes that likely induce specific bioactivities of terpenoids^40,41^. The reference TPSs were enriched with various tailoring enzymes such as cytochrome p450^42^ or short-chain dehydrogenases “adh_short” (Fig. 3d). Therefore, the difference of domain composition between the candidate and reference TPSs may stem from engagement of diverse tailoring enzymes in TPSs.

### Pair-wise alignment of biosynthetic domain architectures in modular biosynthetic gene clusters

While similar BGC machineries can be found across taxonomic superkingdoms, the protein sequences of these BGCs may exhibit variations at sequence-level due to different genomic structures (intergenic and intronic regions) and/or from the rearrangement of protein domains^43^.

**Figure 4:**
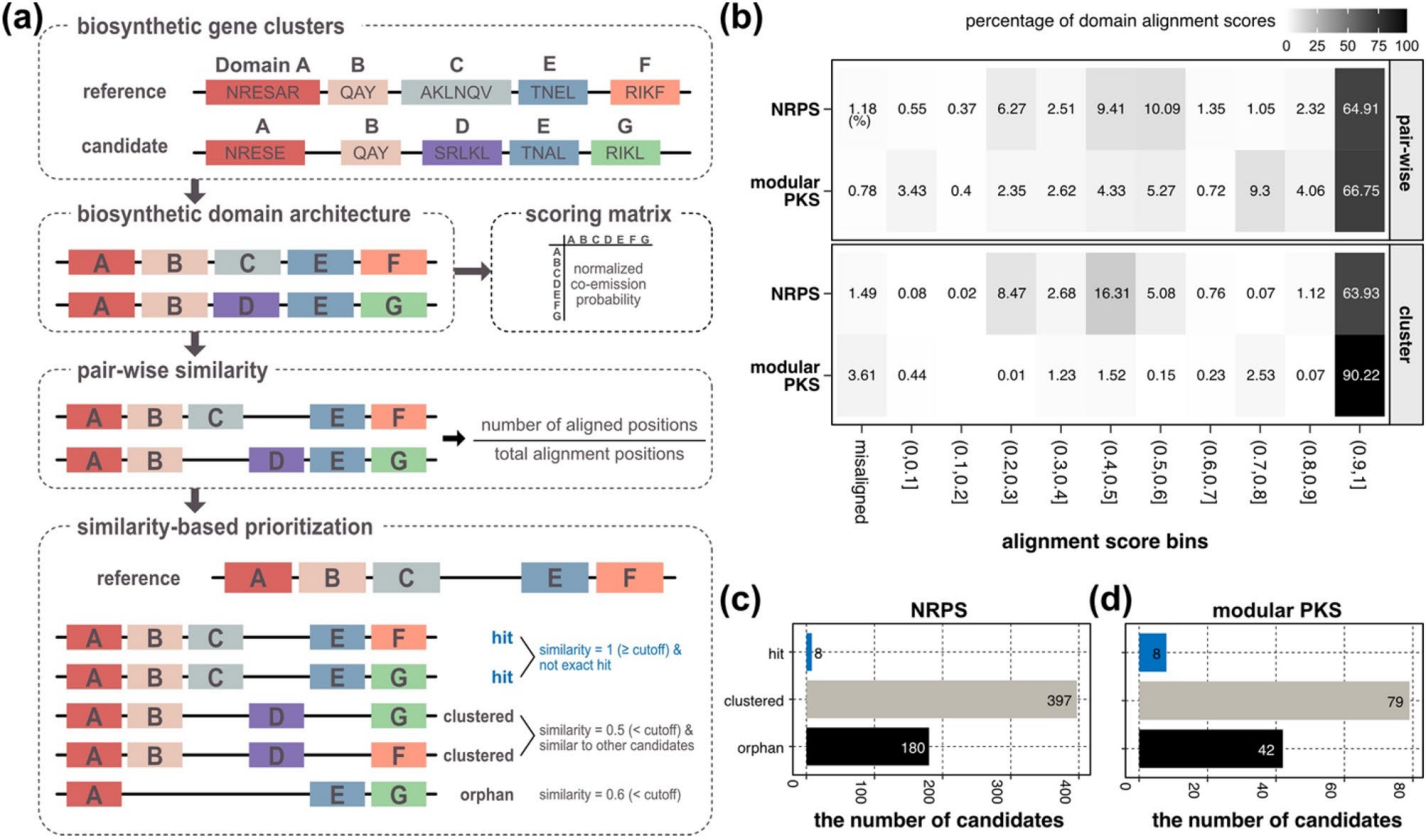
Summary of biosynthetic domain architecture alignment. (**a**) Workflow of biosynthetic domain architecture alignment. (**b**) Distribution of normalized domain-to-domain alignment scores in pair-wise alignment (upper panel) and in cluster alignment (lower panel). (**c**,**d**) The number of “hit”, “clustered”, and “orphan” candidates (**c**) NRPSs and (**d**) modular PKSs.

To mitigate the impact of sequence variations while comparing machineries of biosynthesis based on conserved domains, we assessed biosynthetic domain compositions of BGCs, revealing large variances of domain frequencies even within the same class of BGC (Fig. 3). Unlike non-modular BGCs (type III PKS and TPS), modular BGCs (NRPSs and modular PKSs), operating as a chain of conserved biosynthetic domains, were enriched with a set of domains that often were repeatedly observed more than one time (domain frequency ≥ 1) (Fig. 3a,b). Thus, we (i) vectorized protein sequences to BDA, a sequence of core biosynthetic domains, and (ii) implemented pair-wise BDA alignments between all modular BGCs (Fig. 4a).

By individually comparing each reference and candidate BGC, we selected candidates with similar core biosynthetic mechanisms to those of references or other eukaryotic algal BGCs (Fig. 4a).

#### Alignment scoring matrix

Similar to the amino acid substitution matrices used in sequence alignment problems^44^, we constructed a domain-to-domain alignment scoring matrix by estimating homologies between two pHMMs for each pair of biosynthetic domains. We first estimated the co-emission probabilities between two pHMMs^45^ and then normalized them (Fig. 4a). The normalized co-emission probability of 1 refers to the complete domain-to-domain homology, while a value close to 0 refers to weak homology (Fig. 4b). Homology scores for the domain pairs with zero co-emission probabilities were set to -1 to prevent misalignment (Fig. 4b).

#### Biosynthetic domain architecture alignment

Using the domain alignment scoring matrix, we performed pair-wise BDA alignments that aim for global homology of each pair of BDAs^46,47^. From each pair-wise BDA alignment, we calculated BDA similarity as the proportion of the number of aligned domain loci to the total length of alignment (Fig. 4a). As a result, we generated pair-wise BDA similarity scores between all candidate and reference BGCs. In all pair-wise BDA alignment, we observed that 1.18% (NRPS) and 0.78% (modular PKS) of domain pairs were misaligned, which accounts for domain pairs with zero co-emission probability aligned at the same position (Fig. 4b).

### Clustering based on biosynthetic domain architecture similarity

**Figure 5:**
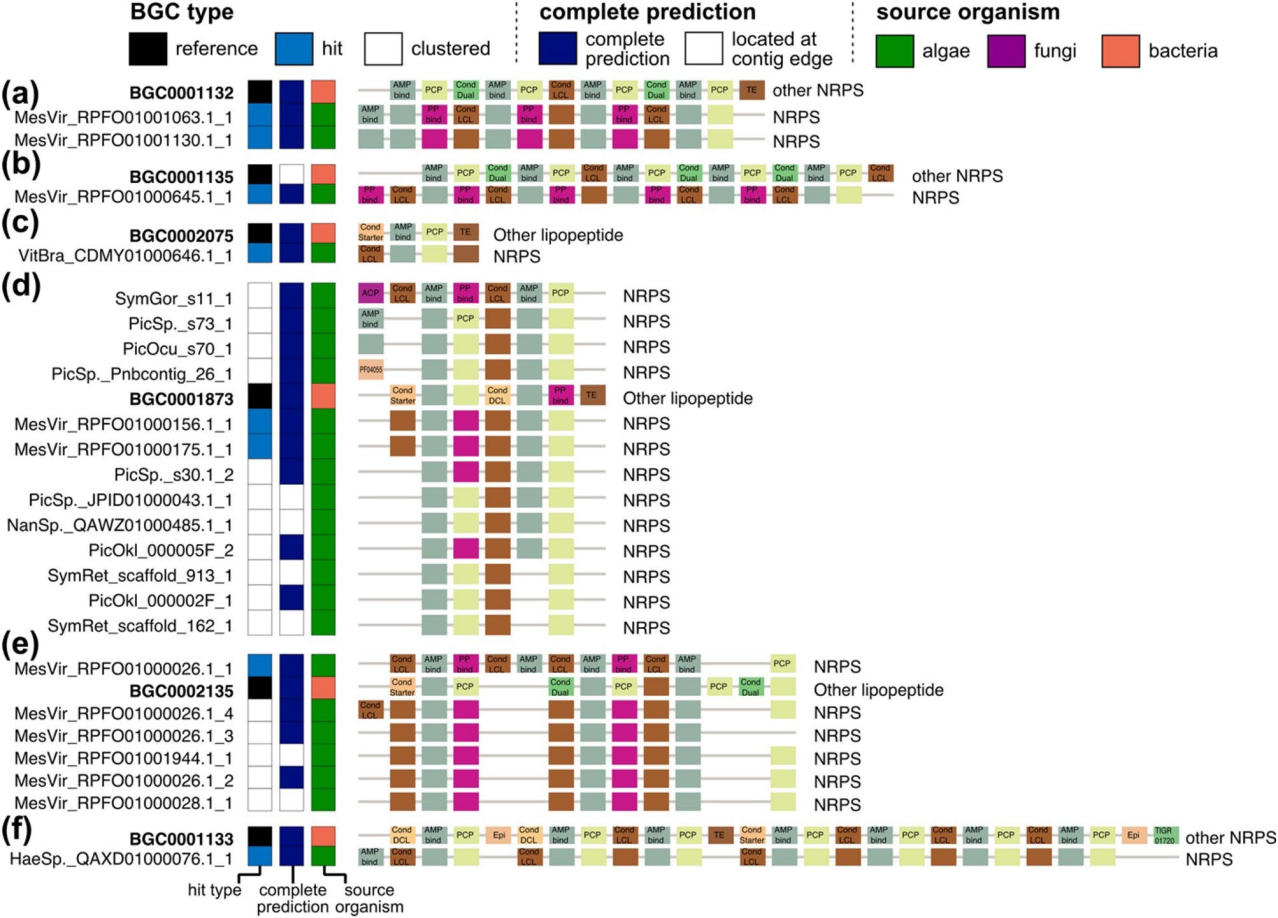
Biosynthetic domain architectures of the candidate NRPSs similar to the reference NRPSs. (**a**–**f**) clusters including candidate NRPSs sharing domain architectures with (**a**) BGC0001132, (**b**) BGC0001135, (**c**) BGC0002075, (**d**) BGC0001873, (**e**) BGC0002135, and (**f**) BGC0001133. Subclass annotations are marked on the right of biosynthetic domain architectures. Biosynthetic domains appearing repeatedly at the same loci are omitted. Domain symbols are summarized in Table S4.

Based on pair-wise BDA similarities, we clustered BGCs with BDAs that were at least 80% similar (BDA similarity ≥ 0.8) (Fig. 4a). Based on BDA similarities to the reference BGCs, candidate BGCs were further categorized as “orphan”, “clustered”, and “hit” candidates (Fig. 4a). Hit candidates refer to the candidates of which BDAs have at least 80% similarity to BDAs of the references. Clustered candidates refer to the candidates that are not similar to BDAs of the references but similar to BDAs of the other candidates. Orphan candidates refer to the candidates of which BDAs are not similar to any other candidates or references. The “hit” and “clustered” candidate BGCs were visualized as clusters, then we selected a total of 16 “hit” candidate BGCs with BDAs similar to the reference BGCs (Fig. 4c,d, and Table 1). We observed higher percentages of misalignments (1.49% in NRPS; 3.61% in modular PKS) in the cluster BDA alignments compared to the pair-wise BDA alignment (Fig. 4b). As we collapsed various BDAs into a single cluster as in Fig. 5, near-end positions of the cluster alignments may contain erroneous domain-to-domain alignment.

**Table 1:** List of “hit” candidate modular biosynthetic gene clusters.

BGC class	Candidate BGC	Similar reference BGC	Source organism
NRPS	MesVir_RPFO01001063.1_1	BGC0001132	*Mesostigma viride* NIES296CAAS
NRPS	MesVir_RPFO01001130.1_1	BGC0001132	*Mesostigma viride* NIES296CAAS
NRPS	HaeSp._QAXD01000076.1_1	BGC0001133	*Haematococcus* sp. NG2
NRPS	MesVir_RPFO01000645.1_1	BGC0001135	*Mesostigma viride* NIES296CAAS
NRPS	MesVir_RPFO01000156.1_1	BGC0001873	*Mesostigma viride* NIES296CAAS
NRPS	MesVir_RPFO01000175.1_1	BGC0001873	*Mesostigma viride* NIES296CAAS
NRPS	VitBra_CDMY01000646.1_1	BGC0002075	*Vitrella brassicaformis*
NRPS	MesVir_RPFO01000026.1_1	BGC0002135	*Mesostigma viride* NIES296CAAS
Modular PKS	AmoSp._RXOD01004016.1_1	BGC0000046	*Amoebophrya* sp. AT52
Modular PKS	ChlSp._QAXI01000426.1_2	BGC0000046	*Chloroidium* sp. JM
Modular PKS	PicOcu_s124_1	BGC0000046	*Picochlorum oculate* UTEXLB1998
Modular PKS	PicSp._s68_1	BGC0000046	*Picochlorum* sp. *soloecismus* DOE101
Modular PKS	NanOce_CP044587.1_1	BGC0001160	*Nannochloropsis oceanica* BR2
Modular PKS	ChlSp._0000218_1	BGC0001340	*Chloroidium* sp. UTEX3007
Modular PKS	ChlSp._QAXJ01000016.1_1	BGC0001340	*Chloroidium* sp. CF
Modular PKS	SymRet_scaffold_258_1	BGC0001340	*Symbiochloris reticulata* SAG5387

#### NRPS

A total of eight “hit” candidate NRPSs had BDAs that are similar to six bacterial reference NRPSs (Fig. 5 and Table 1). These “hit” candidate NRPSs contained BDAs with multiple NRPS elongation modules: condensation–adenylation–carrier protein (Fig. 5). BDAs of the “hit” candidate NRPSs lack thioesterase “TE” that is required to release the synthesized molecule at the end of NRP biosynthesis^48^ (Fig. 5a,d). Some candidates shared an additional starter module of NRPS in the upstream of elongation modules (Fig. 5d). Starter condensation domain “Cond Starter”, condensation/epimerization domain “Cond Dual”, condensation ^D^C_L_ domain “Cond DCL”, and condensation ^L^C_L_ domain are phylogenetically distinguishable^49^. While the bacterial reference NRPSs contained diverse subtypes of condensation domains, eight “hit” candidates and other “clustered” candidates were mainly enriched with condensation ^L^C_L_ (Fig. 5).

Interestingly, no candidate NRPS was similar to the fungal reference NRPSs (Fig. 5). This could have resulted from the majority of fungal reference NRPSs being NRPS-PKS hybrids (16 of 26) that are enriched with iterative KS (Table S2). As the candidate BGCs with iterative KSs were classified as modular PKS, these fungal reference NRPS-PKS hybrids were compared with the candidate modular PKSs (Table S3). In addition, there were enzymes only found in the fungal reference NRPSs, such as tryptophan dimethylallyltransferase “dmat” that catalyzes the first step of the ergot alkaloid pathway^50^, which distinguishes BDA of algal NRPSs and of fungal NRPSs.

A total of 287 among 405 “hit” and “clustered” candidate NRPSs shared exactly same BDAs with at least one other candidate (Fig. S6). Cluster alignments of the “clustered” candidate NRPSs indicated that eukaryotic algal NRPSs were largely grouped into NRPS and NRPS-PKS hybrid. The former group mostly contained one or more of NRPS elongation modules (condensation–adenylation–carrier protein) with NRPS starting module (condensation–adenylation–carrier protein) (Fig. S6). However, many of these candidate NRPSs contained incomplete modules missing adenylation domain and/or condensation domain (Fig. S6). The latter group contained a NRPS starter module (adenylation–carrier protein) and a following hybrid KS module (hybrid KS–acyltransferase–carrier protein) with additional ketoreductase or thioesterase domains (Fig. S6). NRPS-PKS hybrids often contained Coenzyme A ligase “CAL”, one of the sister subfamilies of adenylation domain (Fig. S6a)^51^. In this regard, a handful of NRPS adenylation domains were reported to show Coenzyme A ligase activity^52^. In particular, all of 12 long “clustered” candidate NRPSs (containing ten or more domains) appeared to be NRPS-PKS hybrids, containing more than one PKS module including hybrid KS or modular KS (Fig. S6b). Interestingly, these long “clustered” candidate NRPSs were missing acyltransferase within their PKS modules (Fig. S6).

#### Modular PKS

**Figure 6:**
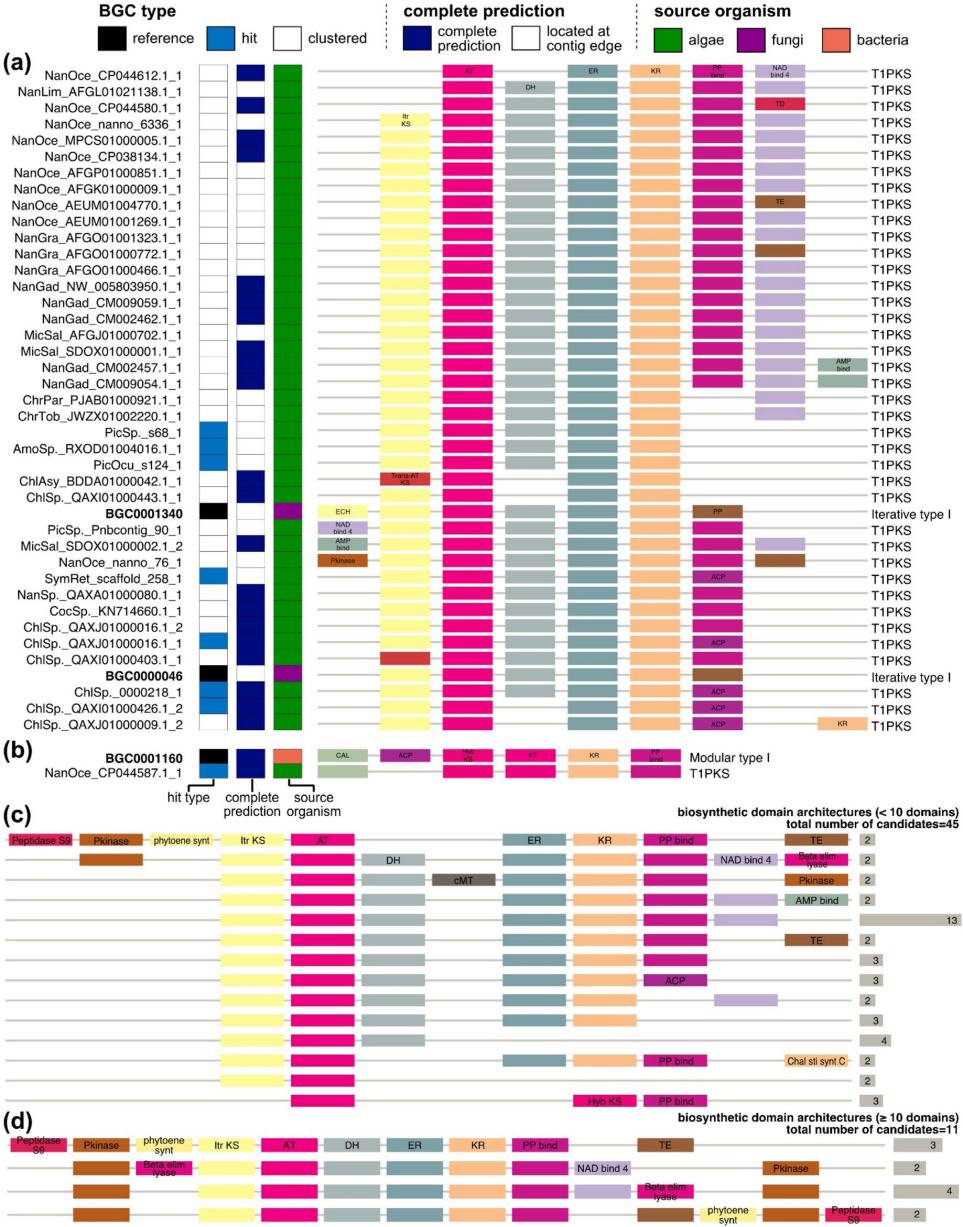
Biosynthetic domain architectures of the candidate modular PKSs similar to the reference modular PKSs. (**a**,**b**) clusters including candidate modular PKSs sharing domain architectures with (**a**) BGC0001340, BGC0000046 and (**b**) BGC0001160. Subclass annotations are marked on the right of biosynthetic domain architectures. (**c**,**d**) Biosynthetic domain architectures detected in at least two of the “clustered” candidate modular PKSs containing (**c**) less than ten domains or (**d**) ten or more domains. Biosynthetic domains appearing repeatedly at the same loci are omitted. Domain symbols are summarized in Table S4.

A total of eight “hit” candidate modular PKSs had BDAs that were similar to two fungal iterative PKSs and one bacterial hybrid PKS (Fig. 6a,b, and Table 1). Four of these eight “hit” candidates contained a canonical iterative PKS module of iterative KS domain–acyltransferase–carrier protein with tailoring enzymes that modify the polyketide product such as dehydratase, enoylreductase, and ketoreductase (Fig. 6a). Three other “hit” candidates (AmoSp._RXOD01004016.1_1, PicOcu_s124_1, and PicSp._s68_1) had iterative PKS module missing a carrier protein (Fig. 6a). One of the fungal reference modular PKS (BGC0001340) contained an additional enoyl-coenzyme A hydratase/isomerase “ECH” in the upstream of the module. One NRPS-PKS hybrid “hit” candidate was found (Fig. 6b), but it was missing a carrier protein between Coenzyme A ligase and hybrid PKS module.

A total of 56 among 70 “clustered” candidate modular PKSs shared BDAs with at least one other candidate (Fig. 6c,d). Among them, a total of 53 candidates had BDAs of a canonical iterative PKS module with various tailoring enzymes, whereas other 3 candidates had incomplete a hybrid PKS module (Fig. 6c). Accordingly, the most abundant BDA of eukaryotic algal modular PKSs was iterative KS–acyltransferase–dehydratase–enoylreductase–ketoreductase–PP-binding carrier protein–nicotinamide adenine dinucleotide-binding domain “NAD bind 4” (n = 13) (Fig. 6c). Protein kinase “Pkinase” were frequently found in the upstream of the candidate iterative PKSs (Fig. 6c,d).

### Transporter genes in biosynthetic gene cluster and its proximate regions

Transporter genes are used to identify natural products with antimicrobial properties^53^, as they facilitate the passage of bioactive compounds out of the cell and into the environment, especially in bacterial examples where the transporter is often co-located with the biosynthetic gene cluster (BGC) in the linear genome sequence^53,54^. Leveraging Pfam^55^ and Transporter Classification Database (TCDB)^56^, we characterized abundant transporter genes in eukaryotic algal BGCs and their 5′ and 3′ proximate regions (20 kb). For this analysis, we analyzed the “complete” candidate BGCs of all four BGC classes that were not located at contig edge (Fig. 2c).

**Figure 7:**
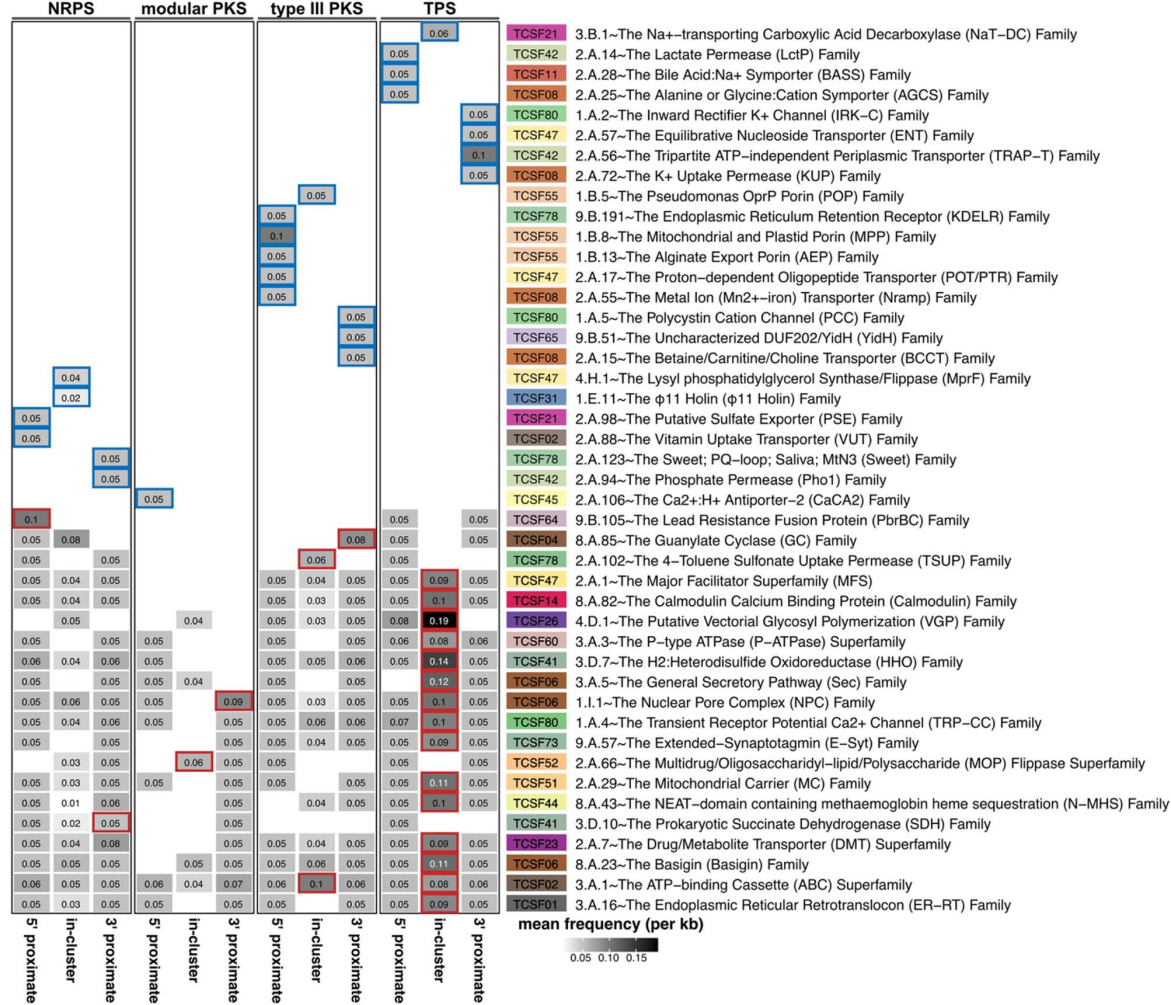
TCDB transporter families enriched in at least one biosynthetic gene cluster class and region. The left panel indicates mean frequency of each TCDB transporter family at one location. Each column of the left panel indicates either of 5′ proximate 20 kb region “5′ proximate”, biosynthetic gene cluster region “in-cluster”, and 3′ proximate 20 kb region “3′ proximate”. Mean frequencies were averaged by every 1 kb non-overlapping window of a region. Red highlight indicates that a transporter family is significantly enriched in the region compared to other regions (Wilcoxon rank-sum test *p*-value < 0.05). Blue highlight indicates that a transporter family is only found in the region. The right panel indicates TCDB family (text) and corresponding superfamily (colored rectangle).

The majority of complete candidate BGCs included at least one transporter gene either in the BGC region “in-cluster” or the proximate regions “proximate”. Specifically, we found at least one transporter gene in 331 of 341 complete candidate NRPSs, 61 of 70 complete candidate modular PKSs, 254 of 355 complete candidate type III PKSs, and 520 of 673 complete candidate TPSs. Notably, we found “in-cluster” transporter genes in 262 candidate NRPSs, 30 candidate modular PKSs, 83 candidate type III PKSs, and 96 candidate TPSs. After compiling the top 10 abundant transporter families for each BGC class, only significantly enriched TCDB transporter families (Wilcoxon rank-sum test *p*-value < 0.05) were shown in Fig. 7. The TCDB classification of families and superfamilies used in this study is detailed in the Table S5.

Certain transporter families, involved in cellular stress and secretion of substrates, were abundant across all BGC classes, such as Endoplasmic Reticular Retrotranslocon Family (3.A.16)^57^, ATP-binding Cassette transporter family (3.A.1)^58^, and Basigin Family (8.A.23)^59^. Notably, a small subset of “in-cluster” transporter families enriched in the candidate NRPSs (n = 2), modular PKSs (n = 1), and type III PKSs (n = 3), whereas 16 transporter families were enriched in the candidate TPS region including globally enriched transporters such as ATP-binding Cassette transporter family (Fig. 7). This disparity of mean frequency of transporter genes comes from modular BGCs spanning longer regions than non-modular BGCs (Fig. 2d). However, it may also indicate that TPS necessitates substrate transport systems, as suggested in a previous study^37^. Our result suggests that BGCs and their proximate regions are enriched with diverse transporters that have not been characterized in the natural product studies. While their operations are still actively studied, many questions remain. This survey offers general guidance on the location and specificity of transporter genes in regards to eukaryotic BGCs.

## Discussion

Despite the genetic potential of eukaryotic algal species for natural product-based drug discovery, the majority of eukaryotic algal BGCs remain largely uncharacterized^15^. The goal of this study was to identify and catalog a spectrum of diverse eukaryotic algal BGCs, laying the foundation for further experimental realization. We successfully leveraged an extensive set of various eukaryotic algal genomes and detected 2476 candidate BGCs. To perform comparative analysis of these structurally diverse BGCs, we vectorized biosynthetic domains into BDA instead of focusing on protein sequences themselves. For the pair-wise alignment of BDAs, we implemented domain-to-domain homology using pHMM comparisons, allowing comparison of BDAs containing homologous biosynthetic domains with sequence-level variations across the broad phylogenetic spectrum of our dataset^45^. Consequently, we identified (i) commonly observed BDAs among the eukaryotic algal BGCs and (ii) promising candidate BGCs with BDAs shared by the MIBiG reference BGCs. Our approach provides an effective means for the comparative analysis of modular BGCs across diverse organisms.

In cases where structural annotation of a genome sequence is absent, antiSMASH relies on fast yet inaccurate ab initio gene prediction^60^, which may lead to false annotations of biosynthetic domains. To address this problem, we utilized a recently published resource to substantiate structural annotations for the unannotated eukaryotic algal genomes^14^. Subsequently, we pre-processed these annotations to reduce false detection in antiSMASH analysis. We recognize that the continuity of genome assemblies may have impacted the quality of BGC detection, as four genome assemblies that failed to yield any BGCs have contig N50 values falling below the bottom 25% of our dataset (Table S1). Modular BGCs, often spanning multiple kilobases, exhibited higher percentages of incomplete detection, possibly influenced by the continuity of genome assemblies (Fig. 2c).

From the MIBiG database, we obtained 308 reference BGCs that are complete and experimentally characterized. These reference BGCs, mainly consisting of modular BGCs (Fig. 2a), exhibited significantly larger sizes and more numbers of biosynthetic domains compared to the candidate BGCs (Fig. 2d,e). Notably, most of the reference BGCs belonged to bacterial origins (Fig. 2b), indicating phylogenetic distinction from the candidate BGCs. Accordingly, we observed large deviations in the frequencies of biosynthetic domains within each BGC class (Fig. 3), concurring with the large variance and a number of outliers in the size of modular BGCs (Fig. 2d,e). The natural variation of biosynthetic machineries between diverse organisms could have also contributed to the structural variability in modular BGCs. For example, assembly-line PKSs with multiple modules are commonly found in bacteria, whereas fungal PKSs predominantly feature of a single module of iterative PKS^61^. To facilitate a comparative analysis of modular BGCs under the structural variability, we employed the vectorization of biosynthetic domains, a successful approach previously adopted in BGC clustering^21,23^.

Using the vectorization, we generated BDA to represent sequence of each BGC, allowing us to mitigate variations in sequence homology commonly observed in a wide range of organisms. The distinctive feature of the pair-wise BDA alignment lies in the alignment scoring matrix, which is generated using domain homology of antiSMASH pHMMs^16^. This process incorporated sequence homology of different biosynthetic domains into the pair-wise BDA alignment, placing emphasis on the domain-level conservation of BGCs rather than sequence-level conservation. The domain homology-derived alignment scoring matrix effectively suppressed misalignment of non-homologous domains (Fig. 4b). In conclusion, the pair-wise BDA alignment estimates structural conservation of modular BGCs between phylogenetically distant organisms.

Employing BDA similarities derived from the pair-wise BDA alignment, we performed clustering of all modular BGCs. Only a handful of eukaryotic algal modular BGCs had BDAs similar to the reference modular BGCs (Fig. 4c,d). The scarcity of “hit” candidate modular BGCs could be attributed to (i) the lack of eukaryotic modular BGCs in the MIBiG database (Fig. 2b) and (ii) the inherent high modularity of these modular BGCs (Fig. 3a,b). The “hit” candidate BGCs demonstrated different compositions of biosynthetic domain subfamilies from the reference BGCs of similar BDAs. For example, eukaryotic algal NRPSs primarily contain condensation ^L^C_L_ domain, whereas condensation ^D^C_L_/Dual were abundant in bacterial NRPSs (Fig. 5). In addition, BDAs of eukaryotic algal modular PKSs showed an abundance of iterative PKS in eukaryotic algae, similar to fungal iterative PKSs (Fig. 6). These findings support that the key biosynthetic machineries of non-model eukaryotes are shared by those of bacteria in different forms.

The multi-modularity of NRPSs likely contributes to low BDA similarities, due to the repetitive occurrence of NRPS elongation modules or NRPS-PKS hybrid modules (Fig. S6b). A notable example includes an eukaryotic algal NRPS-PKS hybrids (SymGor_s2_6) with 85 biosynthetic domains spanning 124 kb and a bacterial reference NRPSs (BGC0000967) with 143 biosynthetic domains spanning 148 kb. This aspect highlights a limitation in our approach that requires improvement for more inclusive analysis of BDAs. Despite this limitation, our pair-wise BDA similarity has proven effective in handling sequence-level variations across diverse organisms while still capturing conservations of the key biosynthetic machineries in modular BGCs.

The current version of antiSMASH offers two types of comparative analysis for BGCs: one based on sequence-level homology, known as “ClusterBlast”^62^, and another based on the vectorization of biosynthetic domains and gene synteny, known as “ClusterCompare”^16^. ClusterBlast, sensitive to the sequence identities, did not find any MIBiG BGCs similar to our candidate modular BGCs. On the other hand, ClusterCompare incorporates the vectorization biosynthetic domain as well as sequence identity of domains, similar to BiG-SCAPE^21^.

For example, ClusterCompare suggested BGC0001132 as the top hit for the “hit” candidate NRPS (MesVir_RPFO01001063.1_1) (Fig. 5a, Fig. S7, and Table 1), whereas it yielded a discordant result from our result for the other “hit” candidate (MesVir_RPFO01000156.1_1) (Fig. 5d, Fig. S8, and Table 1). MesVir_RPFO01000156.1_1 has a BDA of condensation ^L^C_L_–adenylation–PP-binding acyl carrier protein–condensation ^L^C_L_–adenylation–peptidyl carrier protein, which is similar to the BDA of BGC0001873 (condensation starter–adenylation–peptidyl carrier protein–condensation ^D^C_L_–adenylation–PP-binding acyl carrier protein–thioesterase) (Fig. 5d). However, ClusterCompare selected BGC0000342 as the top hit for MesVir_RPFO01000156.1_1, which contains three additional domains (condensation ^D^C_L_–adenylation–peptidyl carrier protein–condensation ^L^C_L_–adenylation–nitrogen methyltransferase “nMT” –peptidyl carrier protein–peptidyl carrier protein–condensation ^D^C_L_) (Fig. S9).

This discrepancy arises from the difference in incorporating sequence identity of biosynthetic domains into the comparative analysis. Our pair-wise BDA alignment performs BDA alignment using pHMM-to-pHMM similarity as alignment scores, allowing a homologous domain match for functionally similar domains (e.g., peptidyl carrier protein and acyl carrier protein). Thus, our method emphasizes the conservation in the vectorized biosynthetic domains. In contrast, ClusterCompare pursues the conservation of sequence-level similarity in each of vectorized biosynthetic domains. This aspect of ClusterCompare allows for a stringent comparison of BGCs within close phylogenetic realms but may impede comparisons between phylogenetically distant species. For example, ClusterCompare similarity score between two similar eukaryotic BGC and bacterial BGC (MesVir_RPFO01001063.1_1 and BGC0001132 similarity = 0.24, Fig. S7) was lower than that of two bacterial BGCs (NC_004808.2 and BGC0001100 similarity = 1.81, see Fig. 3 of Blin et al.^16^). ClusterCompare currently supports the older version of the reference BGCs from MIBiG v2.0 and cannot be customized to specifically target experimentally characterized BGCs.

This study introduces a comprehensive array of diverse eukaryotic algal BGCs, employing a cutting-edge tool in conjunction with a novel strategy for comparative analysis of BGCs. Our pair-wise BDA alignment was specifically designed for the comparative analysis of structurally complex BGCs across diverse organisms. Hence, it allows us to harness the full potential of the MIBiG BGCs despite their phylogenetic disparity from our dataset. We believe this study serves as a noteworthy example of BGC characterization in a broad spectrum of non-model eukaryotes, providing a valuable resource for the realization of novel natural product-based drugs.

## Materials and methods

### Genome sequences and annotation

We retrieved algal genome assemblies using the query “eukaryotic algae” from public genome databases, including the NCBI GenBank assembly database^63^ and the JGI database^64^ (Table S1). We used the Entrez tool^65^ implemented in the Biopython package^66^ to retrieve data from the NCBI GenBank assembly database (accessed July 30, 2020). Genome assemblies from other public genome databases were individually obtained (Table S1). Genome annotations and protein sequences corresponding to the genome assemblies were also retrieved (Table S1).

First, we excluded genome assemblies that are incomplete or sequenced from unreliable sources. We also excluded genome annotations with (i) anomalies in core annotation features and/or (ii) discordances between genome assembly, structural annotation, and protein sequences (Table S1). Next, we performed BUSCO analysis v4.0.6^67^ to assess the quality of the genome assembly (-m genome) and annotation (-m protein) for downstream analyses. For each genome, BUSCO dataset of OrthoDB v10^28^ was selected using automatic lineage selection enabled with BUSCO genome mode (--auto-lineage-euk, -m genome) (Table S1). We excluded genome assemblies with BUSCO genome missing rates (-m genome) over 75%. Additionally, we used protein sequence sets extracted from annotations to exclude annotations with BUSCO protein missing rates (-m genome) over 50%. Subsequently, we performed gene prediction for the genome sequences without annotations, using Braker v2.1.6^27^ according to Kwon et al.^14^. Detail of the gene prediction process is summarized in Text S2. Detail of the dataset is summarized in Table S1.

### Computational detection of candidate biosynthetic gene clusters

After the data filtering step, we selected the longest isoforms per each gene in structural annotations using *agat_sp_keep_longest_isoform.pl* of AGAT v0.8.0 package^68^. Subsequently, we also checked the validity of the structural annotations using antiSMASH v6.1.1^16^. In the case of the validity check failing due to overlapping exons in the structural annotation, we merged overlapping loci using *agat_convert_sp_gxf2gxf.pl* of AGAT package (--mergi_loci). In case of the validity check failing due to mixed stranded features in a transcript, we fixed strand information of the features using an in-house python script. Lastly, we used the modified structural annotations for antiSMASH and excluded the annotations that failed to pass. For three genomes with failed annotations, we used Braker2 predicted gene sets. For each genome sequence and corresponding annotation data, we performed antiSMASH with fungiSMASH “--taxon = fungi”, ClusterCompare “--cc-mibig”, and ClusterBlast “--cb-knownclusters” parameters enabled.

We used an in-house python script to parse Genbank format outputs of antiSMASH. After excluding BGCs on the contigs shorter than 10 kb, we classified candidate BGCs into BGC classes and subclasses using “product” labels that were predicted by antiSMASH (see *BGC subclass* column of Table S3): “NRPS” and “NRPS-like” subclasses were grouped into NRPS class; “T1PKS” and “T2PKS” subclasses were grouped into modular PKS class; “T3PKS” subclass was annotated as type III PKS class; “terpene” subclass was annotated as TPS class.

### Experimentally characterized biosynthetic gene clusters

We retrieved reference BGCs from the MIBiG database v3.1^15^. Among a total of 2502 MIBiG entries, we selected 427 BGCs that are annotated as “complete” in MIBiG JSON data and are supported by any experimental evidence. Chemical activity and compound information of each reference BGC was parsed from “chem_acts” and “compound” features of MIBiG JSON data. Similar to the candidate BGCs, the reference BGCs were grouped into four major BGC classes based on both class (“biosyn_class”) and subclass (“subclass”) annotation of MIBiG JSON data. “NRP” BGCs were classified as NRPS class. “Polyketide” BGCs including PKS subclasses other than “type III” subclass were classified as modular PKS class. “Polyketide” BGCs with only “type III” subclass were classified as type III PKS class. “Terpene” BGCs were classified as TPS class. Class labels of the reference BGCs were not exclusive.

### Post-processing of biosynthetic gene clusters

To extract biosynthetic domain information, we parsed GenBank format outputs of antiSMASH for all candidate and reference BGCs. We selected biosynthetic domain information that were annotated with “NRPS_PKS” and/or “sec_met_domain” CDS annotation features. In case of loci where both of these two annotation features exist, we selectively parsed one over the other feature based on class of the BGC; we primarily parsed “NRPS_PKS” in NRPS and modular PKS classes while primarily parsing “sec_met_domain” in type III PKS and TPS classes. We trimmed BGC regions to only include CDSs that contain biosynthetic domains. Strand information and coordinates were summarized according to the trimmed BGC region (Tables S2 and S3).

### Biosynthetic domain composition and architecture

As described in the post-processing step, we summarized a sequence of biosynthetic domains in each BGC. For each BGC class, the compositions of biosynthetic domains were summarized by the average frequency of each biosynthetic domain. We selected and visualized the ten most frequently detected biosynthetic domains for candidate BGCs and reference BGCs, respectively. For modular BGCs (NRPS and modular PKS), we generated a sequence of biosynthetic domains for each BGC, or BDA, which transforms protein sequences into a simplified form composed of labels of protein domain.

### Pair-wise biosynthetic domain architecture similarities

We estimated pair-wise BDA similarities between all pairs of candidates and references of each BGC class. To provide the alignment scoring matrix for the alignment program MAFFT, we used similarities between every two biosynthetic domains by comparing HMM profiles of those domains^45^. We extracted HMM profiles of all biosynthetic domains from antiSMASH v6.1.1 data and then estimated pair-wise similarities of HMM profiles using Profile Comparer v1.5.6^45^, of which detail is summarized in Text S3.

As a result, we generated an alignment scoring matrix with these pair-wise profile HMM similarity scores. We used MAFFT v7.471^69^ alignment tool to generate pair-wise BDA alignments, implementing text alignment (--text and --textmatrix). According to the developer’s suggestion, we set MAFFT alignment parameters to allow global alignment (--globalpair and --allowshift) with gap opening/extending penalty of zero (--op = 0, --gop = 0, and --ep = 0). To estimate pair-wise BDA similarity, we calculated uncorrected *p*-distance for each pair-wise alignment. Total length (*N*) accounted for the total length of each pair-wise alignment. Any alignment position with biosynthetic domains in both BGCs (alignment positions without gaps) was considered to be match (*M*). Uncorrected *p*-distance was calculated as 1 − *M*/*N*, therefore, BDA similarity was calculated as *M*/*N*.

### Clustering based on biosynthetic domain architecture similarity

For each BGC class, we generated a pair-wise BDA similarity matrix. Based on the BDA similarity matrix, we clustered all BGCs with similarities of 0.8 or higher. We greedily grouped candidates/references into a cluster where one member has BDA similarity of 0.8 or higher with any of the other members of the cluster. Accordingly, candidate BGCs were classified into three groups: “orphan”, “clustered”, and “hit”. Hit candidate BGCs refer to the candidates of which BDAs are similar to those of the reference BGCs (0.8 ≤ BDA similarity). Clustered candidates refer to the candidates of which BDAs are not similar to BDAs of any reference but similar to BDAs of the other candidates. Orphan candidates refer to the candidate BGCs of which BDAs are not similar to any other candidates or references.

Each BDA cluster was re-aligned for visualization using MAFFT (Fig. 4a). Alignment parameters for multiple BDA alignment were set to the same as those for pair-wise BDA alignment (--globalpair, --allowshift, --op = 0, --gop = 0, and --ep = 0). BDA clusters were visualized using the ggplot2 package^70^ within R^71^.

### Proximate transporter gene search

We searched for transporter genes in the candidate BGCs that were not located at contig edge. Using Pfam-A set v33.1^55^ and HMMER v3.1b2, we annotated transporter family/superfamily from the Transporter Classification Database (TCDB, accessed at February 2021)^56^ with Pfam-A HMM profiles^72^ (Table S5). Detail of the process is summarized in Text S4.

The top 10 most abundant TCDB families per each BGC class were collected. Frequency of each transporter family was calculated per each kilo base pairs; for example, a frequency of 1 indicates one transporter gene found in a 1 kb region. For each TCDB family, we performed Wilcoxon rank-sum test between transporter family frequencies of a region (e.g., 5′ proximate region) of a BGC class (e.g., NRPS) and transporter family frequencies except for the region of the BGC class in R^71^.

### Supplementary Information

Supplementary Information.

Supplementary Table S1.

Supplementary Table S2.

Supplementary Table S3.

## Data Availability

The antiSMASH output files (GenBank format) for candidate BGCs and the visualization (PDF format) of the clusters composed of “clustered” candidate BGCs are available in *Mendeley Data* (reserved DOI: 10.17632/n9dgpr3t7d.1, currently available before publication at https://data.mendeley.com/datasets/n9dgpr3t7d/1.
