# Supplementary material for: Global characterization of biosynthetic gene clusters in non-model eukaryotes using domain architectures
Source: Sci Rep. 2024 Jan 17;14:1534. doi: 10.1038/s41598-023-50095-3 (PMC10794256; doi:10.1038/s41598-023-50095-3)
Supplement: Supplementary file 1 — Supplementary Information. [file 41598_2023_50095_MOESM1_ESM.pdf]

Supplemental Materials

Global Characterization of Biosynthetic Gene Clusters in Non-model Eukaryotes Using Domain Architectures

Taehyung Kwon[1], Blake T. Hovde[1]*

[1] Genomics and Bioanalytics, Bioscience Division, Los Alamos National Laboratory, New Mexico, United States

* Corresponding author

E-mail address: hovdebt@lanl.gov

Supplemental Text

Text S1. Summary of canonical domains in modular BGCs

Text S2. Gene prediction

Text S3. Pair-wise domain similarity

Text S4. Transporter gene search

Supplemental Figures

Figure S1. Summary of five most abundant chemical activities in MIBiG reference BGCs

Figure S2. Correlations of the number of the candidate BGCs and genome size

Figure S3. Correlations of the number of the candidate BGCs and assembly contig N50

Figure S4. Network of biosynthetic domain co-existence in the candidate type III PKS

Figure S5. Network of biosynthetic domain co-existence in the candidate TPS

Figure S6. Frequently detected biosynthetic domain architectures in the clustered candidate NRPSs

Figure S7. ClusterCompare analysis result for MesVir_RPFO01001063.1_1

Figure S8. ClusterCompare analysis result for MesVir_RPFO0100156.1_1

Figure S9. Biosynthetic domain architecture of BGC0000342

Supplemental Tables

Table S4. Summary of biosynthetic domain symbols presented in this study

Table S5. Summary of TCDB classification system presented in this study

Text S1. Summary of canonical domains in modular BGCs

NRPS: In NRPS biosynthesis, adenylation domain plays a key role of recognizing and activating the amino acid substrate, thus it also determines the substrate specificity [1]. PCP domain is a peptidyl-carrier protein mainly found in NRPS and is homologous to other acyl-carrier proteins mostly found in PKSs such as acyl-carrier proteins ("ACP" or phosphopantetheine acyl-carrier proteins "PP" and "PP bind"). These carrier proteins bind to the substrate activated by an adenylation process and pass the substrate to condensation domains [1]. Condensation domains, located at the N-terminus of NRPS elongation modules, catalyze a peptide bond between the product of the upstream module and the amino group of the module [2]. These domains are considered to be canonical in starting modules (e.g., adenylation – PCP) and elongation modules (e.g., condensation - adenylation – PCP) of NRPS.

Modular PKS: Acyltransferase domain loads acyl-CoA metabolites onto the acyl-carrier proteins [3]. Acyl-carrier proteins, mainly found in PKS and similar to PCP domain of NRPS, pass the substrate to KS domain with their phosphopantetheinyl arms [3]. KS domains catalyze a condensation between the previously synthesized polyketide chain and the extender substrate [3]. KS domains determine the assembly process with either in-module acyltransferase (*cis*-AT PKS) or free-standing acyltransferase (*trans*-AT PKS) [4]. *Cis*-AT PKSs are then largely divided into iterative PKS and assembly-line PKS [5]. Iterative PKSs operates with a single module in charge of multiple catalytic cycle, while assembly-line PKSs elongate the polyketide chain over multiple modules [6]. In particular, iterative KS domains, operating iterative PKSs, were known to be distinct from KS domains operating assembly-line PKSs in terms of function and sequence structure [7], as iterative KS domains recapture the substrate after each round of synthesis for polyketide elongation. Distinct from iterative KS, modular KS and hybrid KS cooperate with carrier proteins of the upstream module, as a part of each module of assembly-line PKSs or NRPS-PKS hybrids, respectively [3].

Text S2. Gene prediction

We used Braker2 to annotate coding regions for the genomes without annotations or with low-quality annotations (**Tables S1**). Briefly, we implemented two modes of Braker2: an *ab initio* gene prediction mode (Braker-ES) and gene prediction with protein hints detected using OrthoDB v10.0 (Braker-EP) [8]. Based on the result of a previous study [9], we used Braker-ES for the genomes with BUSCO genome complete rate < 90% and Braker-EP for the genomes with BUSCO genome complete rate ≥ 90%.

For Braker-ES method, we used Braker2 gene prediction with *ab initio* gene finding algorithm GeneMark-ES (--esmode) [10]. We lowered the minimum contig size to 10 Kb (--min_contig=10000) and enabled softmasked region detection for softmasked genomes (--softmasking). For Braker-EP method, we used Braker2 gene prediction with GeneMark-EP/EP+ pipeline including ProtHint, Spaln2, and DIAMOND [11-13]. This GeneMark-EP/EP+ pipeline aligns protein sequences to the genome assembly to find exon-intron boundary hints. First, to generate protein sequence files, we prepared clade-specific orthologous gene sets of the following algal clades by parsing the OrthoDB v10.0 database [8]: Eukaryota, Chlorophyta, Chlorophyceae, Rhodophyta, and Bacillariophyta. Based on the taxonomic classification of each genome, we used a clade-specific orthologous gene set of the most specific clade. For each clade, we collected all protein sequences in OrthoDB v10.0 orthogroups that are shared by at least 80% of the species of the clade. Lastly, we performed Braker2 with protein hint (--prot_seq) and the GeneMark-EP/EP+ pipeline (--epmode) and also used the same parameters as in the Braker-ES (--min_contig=10000 and --softmasking).

Text S3. Pair-wise domain similarity

For each pair of biosynthetic domains, we performed Profile Comparer analysis with global match (-mode global-global). In Profile Comparer output, co-emission probabilities between two HMM profiles (domain X and Y) is a generalized log-odds score representing significance of match between two HMM profiles [14]. Since the co-emission probability of each pair (X and Y) is governed by the length of alignment between X and Y, we used a normalized co-emission probability as an alignment score. We divided the co-emission probability of domain X and Y by the denominator: the larger value among co-emission probability of domain X and X and co-emission probability of domain Y and Y. Therefore, each pair of different domains (X and Y) had an alignment score ranging from 0 to 1. Each pair of same domains (X and X) had 1. In case two domains had zero co-emission probability, -1 was assigned as an alignment score to prevent misalignment.

Text S4. Transporter gene search

We fetched transporter gene sequences from TCDB while assigning TCDB superfamily and family classification for transporter genes based on TCDB classification (**Table S5**). To find homologous sequences to the TCDB transporters, we annotated TCDB transporter genes using Pfam-A [15]. We used hmmfetch and hmmpress within the HMMER to generate Pfam-A HMM profiles [16]. We then annotated transporter genes using hmmsearch of HMMER with Pfam-defined cutoffs (--cut_ga). Based on the Pfam-to-transporter annotation, we assigned TCDB family/superfamily classification to Pfam pHMMs.

We consolidated TCDB families that had at least five hits to any Pfam IDs. During this process, we assigned a TCDB family that represents a Pfam ID the most to the Pfam ID, which is indicated by the highest number of hits. As a result, we generated transporter-specific pHMMs, a subset of Pfam-A pHMMs that are represented by TCDB transporter genes. Next, we annotated all amino acid sequences of each BGC region "in-cluster region" and 5' (20 Kb) and 3' (20 Kb) of the BGC region "5' and 3' proximate region". All protein sequences were annotated using hmmsearch with E-value cutoff of 1e-10 in assumption of database size of 10,000 sequences (-Z 10000).

Supplemental Text References

1. Walsh, C.T. Polyketide and nonribosomal peptide antibiotics: modularity and versatility. *Science* **303**, 1805-1810 (2004).
2. Lautru, S. & Challis, G.L. Substrate recognition by nonribosomal peptide synthetase multi-enzymes. *Microbiology* **150**, 1629-1636 (2004).
3. Nivina, A., Yuet, K.P., Hsu, J. & Khosla, C. Evolution and diversity of assembly-line polyketide synthases: Focus review. *Chemical reviews* **119**, 12524-12547 (2019).
4. Piel, J. Biosynthesis of polyketides by trans-AT polyketide synthases. *Natural product reports* **27**, 996-1047 (2010).
5. Hertweck, C. The biosynthetic logic of polyketide diversity. *Angewandte Chemie International Edition* **48**, 4688-4716 (2009).
6. Crawford, J.M. & Townsend, C.A. New insights into the formation of fungal aromatic polyketides. *Nature Reviews Microbiology* **8**, 879-889 (2010).
7. Yadav, G., Gokhale, R.S. & Mohanty, D. Towards prediction of metabolic products of polyketide synthases: an in silico analysis. *PLoS computational biology* **5**, e1000351 (2009).
8. Kriventseva, E.V. *et al.* OrthoDB v10: sampling the diversity of animal, plant, fungal, protist, bacterial and viral genomes for evolutionary and functional annotations of orthologs. *Nucleic acids research* **47**, D807-D811 (2019).
9. Kwon, T., Hanschen, E.R. & Hovde, B.T. Addressing the pervasive scarcity of structural annotation in eukaryotic algae. *Scientific Reports* **13**, 1687 (2023).
10. Ter-Hovhannisyan, V., Lomsadze, A., Chernoff, Y.O. & Borodovsky, M. Gene prediction in novel fungal genomes using an ab initio algorithm with unsupervised training. *Genome research* **18**, 1979-1990 (2008).
11. Brůna, T., Lomsadze, A. & Borodovsky, M. GeneMark-EP+: eukaryotic gene prediction with self-training in the space of genes and proteins. *NAR genomics and bioinformatics* **2**, lqaa026 (2020).
12. Strassert, J.F., Jamy, M., Mylnikov, A.P., Tikhonenkov, D.V. & Burki, F. New phylogenomic analysis of the enigmatic phylum Telonemia further resolves the eukaryote tree of life. *Molecular biology and evolution* **36**, 757-765 (2019).
13. Iwata, H. & Gotoh, O. Benchmarking spliced alignment programs including Spaln2, an extended version of Spaln that incorporates additional species-specific features. *Nucleic acids research* **40**, e161-e161 (2012).
14. Madera, M. Profile Comparer: a program for scoring and aligning profile hidden Markov models. *Bioinformatics* **24**, 2630-2631 (2008).
15. Mistry, J. *et al.* Pfam: The protein families database in 2021. *Nucleic acids research* **49**, D412-D419 (2021).
16. Eddy, S.R. Accelerated profile HMM searches. *PLoS computational biology* **7**, e1002195 (2011).

Figure S1. Summary of the five most abundant chemical activities in MIBiG reference BGCs

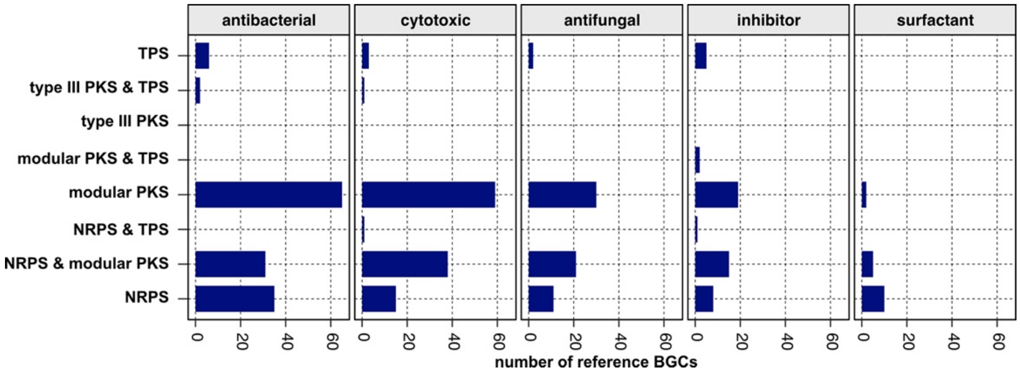

Figure S2. Correlations of the number of the candidate BGCs and genome size

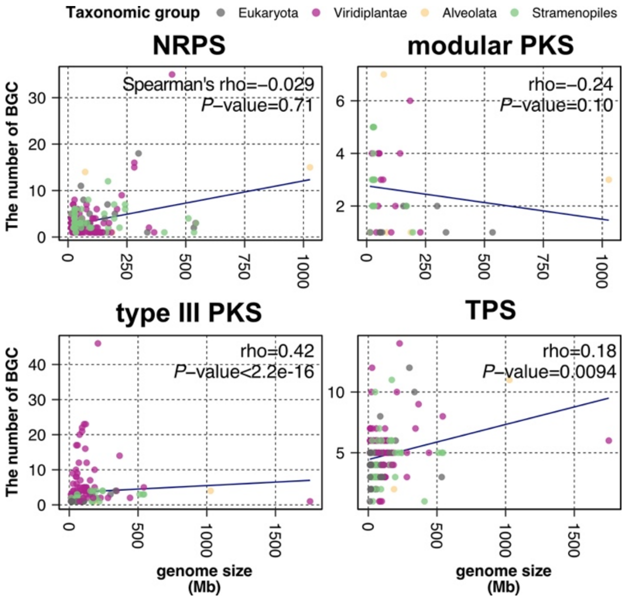

Figure S3. Correlations of the number of the candidate BGCs and assembly contig N50

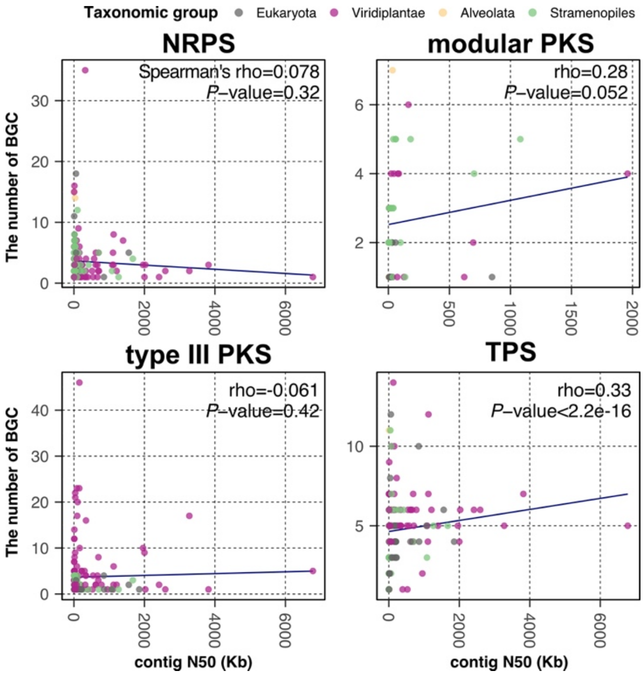

Figure S4. Network of biosynthetic domain co-existence in the candidate type III PKSs

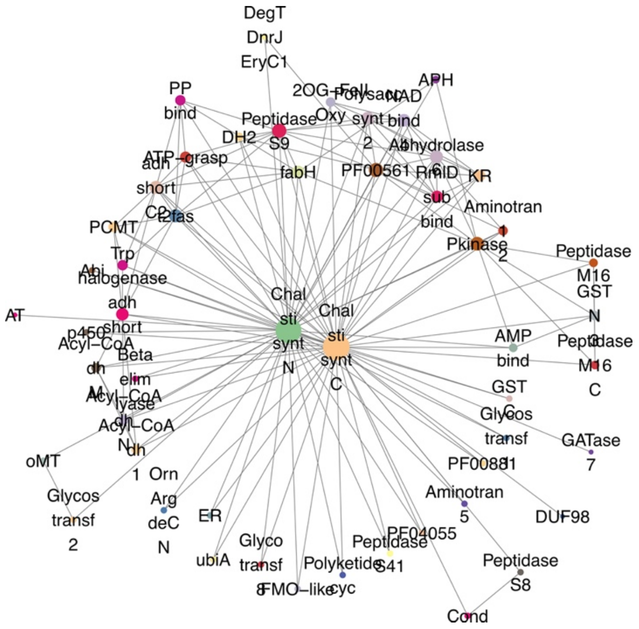

Figure S5. Network of biosynthetic domain co-existence in the candidate TPSs

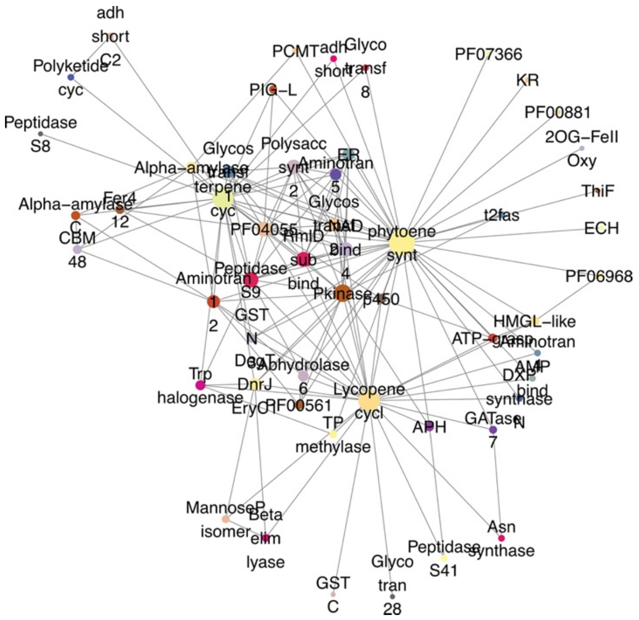

Figure S6. Frequently detected biosynthetic domain architectures in the clustered candidate NRPSs

Only biosynthetic domain architectures detected in at least two of the clustered candidate NRPSs are displayed. (a) Biosynthetic domain architectures with less than ten domains. (a) Biosynthetic domain architectures with ten or more domains.

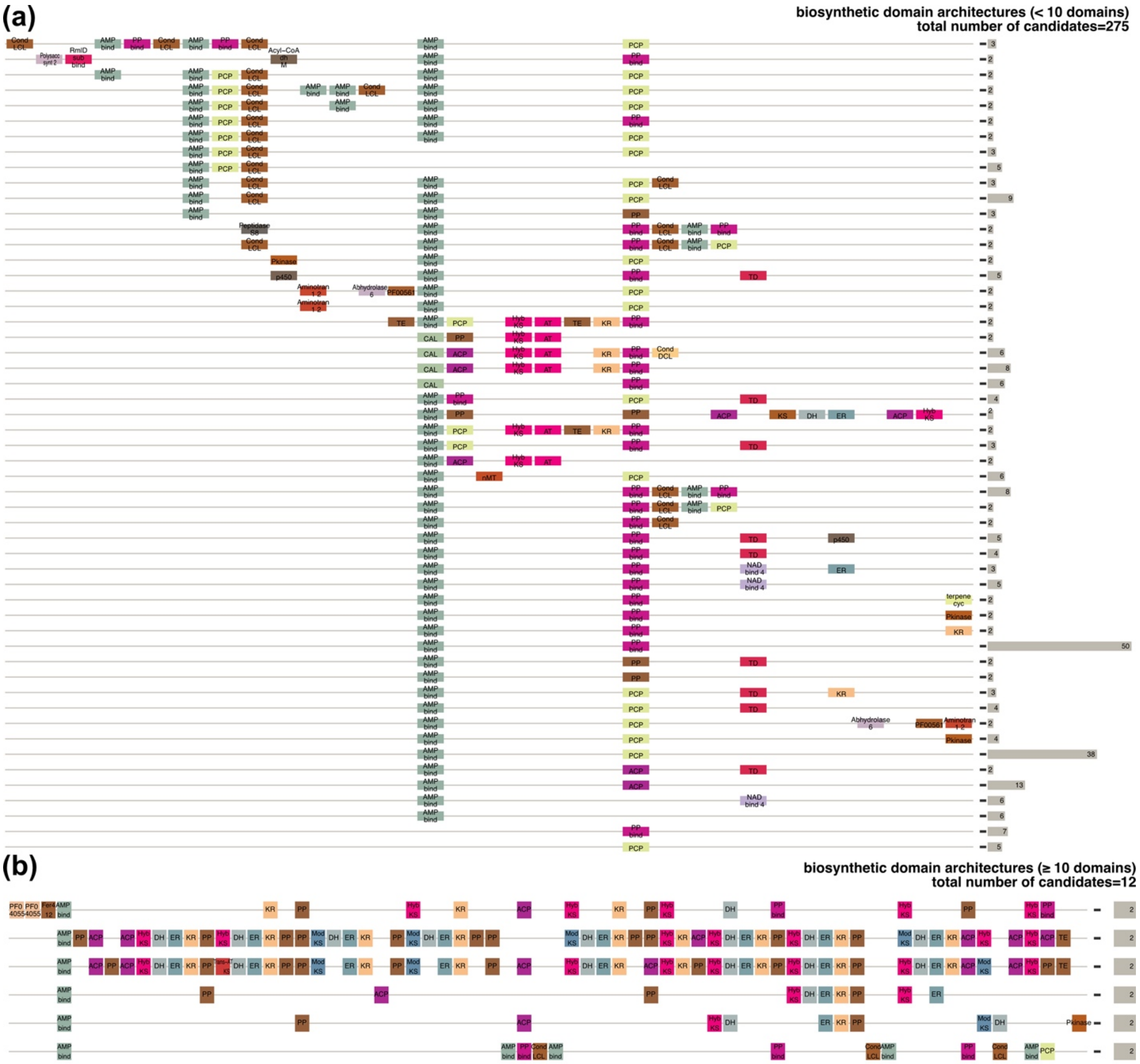

Figure S7. ClusterCompare analysis result for MesVir_RPFO01001063.1_1

The top five MIBiG hits were displayed.

RPFO01001063.1 - Region 1 - NRPS

Location: 130,437 - 198,086 nt. (total: 67,650 nt) Show pHMM detection rules used

Download region SVG | Download region GenBank file

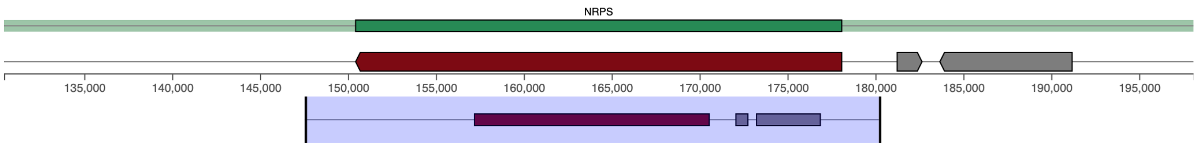

Legend:

core biosynthetic genes | additional biosynthetic genes | transport-related genes | regulatory genes | other genes | resistance

reset view | zoom to selection

NRPS/PKS domains | MIBiG comparison | NRPS/PKS modules

Similar gene clusters

Analysis type: Protocluster to Region

Query

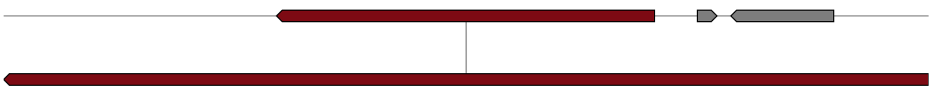

Reference: BGC0001132.1: 0-12417

Reference	NRPS	Similarity score	Type	Compound(s)	Organism
BGC0001132.1		0.24	NRP	xenotetrapeptide	Xenorhabdus nematophila ATCC 19061
BGC0001844.1		0.23	NRP	holrhizin	Paraburkholderia rhizoxinica HKI 454
BGC0001261.1		0.23	NRP	AM-toxin	Alternaria alternata
BGC0001135.1		0.23	NRP	bicornutin A1, bicornutin A2	Xenorhabdus budapestensis
BGC0001128.1		0.23	NRP	luminmide	Photorhabdus laumondii subsp. laumondii TTO1

Figure S8. ClusterCompare analysis result for MesVir_RPFO0100156.1_1

The top five MIBiG hits were displayed.

RPFO01000156.1 - Region 1 - NRPS

Location: 582,703 - 631,795 nt. (total: 49,093 nt) Show pHMM detection rules used

Download region SVG | Download region GenBank file

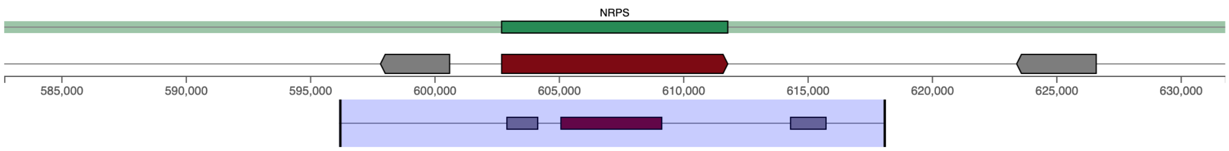

Legend:

core biosynthetic genes | additional biosynthetic genes | transport-related genes | regulatory genes | other genes | resistance

reset view | zoom to selection

NRPS/PKS domains | MIBiG comparison | NRPS/PKS modules

Similar gene clusters

Analysis type: Protocluster to Region

Query

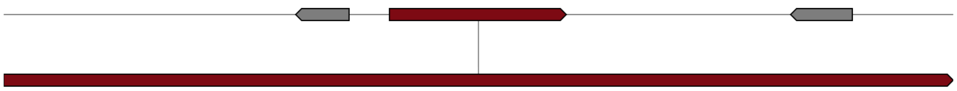

Reference: BGC0000342.1: 1020-10416

Reference	NRPS	Similarity score	Type	Compound(s)	Organism
BGC0000342.1		0.26	NRP	enniatin	Fusarium equiseti
BGC0001833.1		0.24	NRP	icosalide A, icosalide B	Burkholderia gladioli
BGC0001132.1		0.24	NRP	xenotetrapeptide	Xenorhabdus nematophila ATCC 19061
BGC0001825.1		0.23	NRP	xenematide	Xenorhabdus nematophila AN6/1
BGC0001844.1		0.23	NRP	holrhizin	Paraburkholderia rhizoxinica HKI 454

Figure S9. Biosynthetic domain architecture of BGC0000342

Its domain architecture is condensation DCL ~ adenylation ~ peptidyl carrier protein ~ condensation LCL ~ adenylation ~ nitrogen methyltransferase ~ peptidyl carrier protein ~ peptidyl carrier protein ~ condensation DCL.

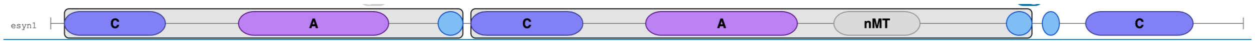

Table S4. Summary of biosynthetic domain symbols presented in this study

Sources denoted as "antiSMASH" indicate profile Hidden Markov Models generated by antiSMASH. Details of the sources are available in https://docs.antismash.secondarymetabolites.org/modules/nrps_pks_domains.

Domain symbol	Description	Source
**Abhydrolase 6**	Alpha/beta hydrolase family	PF12697.6
**ACP**	Acyl-carrier protein domain	Medema et al. 2011
**Acyl-CoA dh M**	Acyl-CoA dehydrogenase, middle domain	PF02770.19
**ADH N**	Alcohol dehydrogenase GroES-like domain	PF08240.15
**adh short**	Enoyl-(Acyl carrier protein) reductase	PF13561.1
**adh short C2**	Enoyl-(Acyl carrier protein) reductase	PF13561.1
**ADH zinc N**	Zinc-binding dehydrogenase	PF00107.29
**AfsA**	A-factor biosynthesis repeat	PF03756.6
**Aminotran 1 2**	Aminotransferase class I and II	PF00155.14
**AMP bind**	Adenylation (AMP-binding enzyme)	PF00501.23
**AT**	Acyltransferase domain	SMART
**Beta elim lyase**	Beta-eliminating lyase	PF01212.23
**Chal sti synt C**	Chalcone and stilbene synthases, C-terminal domain	PF02797.8
**Chal sti synt N**	Chalcone and stilbene synthases, N-terminal domain	PF00195.12
**cMT**	Carbon methyltransferase	Ansari et al. 2008
**Cond DCL**	Condensation domain linking a L-amino acid to a peptide ending with a D-amino acid	Rausch et al. 2007
**Cond Dual**	Dual condensation/epimerisation domain	Rausch et al. 2007
**Cond LCL**	Condensation domain linking a L-amino acid to a peptide ending with a L-amino acid	Rausch et al. 2007
**Cond Starter**	Starter condensation domain	Rausch et al. 2007
**DH**	Dehydratase domain	SMART
**DXP synthase N**	1-deoxy-D-xylulose-5-phosphate synthase	PF13292.7
**ECH**	Enoyl-CoA hydratase/isomerase family	PF00378.15
**Epi**	Epimerization domain	Weber et al. 2009
**ER**	Enoylreductase domain	SMART
**fabH**	β-ketoacyl-acyl carrier protein synthase III	antiSMASH
**Fer4 12**	4Fe-4S single cluster domain	PF13353.4
**fung ggpps2**	Fungal Geranylgeranyl diphosphate synthase	antiSMASH
**Hyb KS**	Hybrid Ketosynthase	Yadav et al. 2009

**Itr KS**	Iterative Ketosynthase	Yadav et al. 2009
**KR**	Ketoreductase	SMART
**Lycopene cycl**	Lycopene cyclase protein	PF05834.5
**Mod KS**	Modular Ketosynthase	Yadav et al. 2009
**NAD bind 4**	Male sterility protein	PF07993.5
**NapT7**	polyprenyl synthase	antiSMASH
**nMT**	Nitrogen methyltransferase	Ansari et al. 2008
**orf2 Ptase**	prenyltransferases	antiSMASH
**p450**	Cytochrome P450	PF00067.17
**PCMT**	Protein-L-isoaspartate(D-aspartate) O-methyltransferase (PCMT)	PF01135.19
**PCP**	Peptidyl-carrier protein domain	Medema et al. 2011
**Peptidase S8**	Subtilase family	PF00082.20
**Peptidase S9**	Prolyl oligopeptidase family	PF00326.19
**PF00561**	alpha/beta hydrolase fold	PF00561.18
**PF04055**	Radical SAM superfamily	PF04055
**phytoene synt**	Phytoene synthase	antiSMASH
**Pkinase**	Protein kinase domain	PF00069.20
**Polysacc synt 2**	Polysaccharide biosynthesis protein	PF02719.10
**PP**	Phosphopantetheine acyl carrier protein group	SMART
**PP bind**	Phosphopantetheine attachment site	PF00550.18
**RmlD sub bind**	RmlD substrate binding domain	PF04321.12
**TD**	Terminal reductase domain	Medema et al. 2011
**TE**	Thioesterase	PF00975.13
**terpene cyc**	Terpene Cyclase	antiSMASH
**TIGR01720**	NRPS-para261: non-ribosomal peptide synthase domain TIGR01720	TIGR01720
**Trans−AT docking**	trans-AT docking domain	Medema et al. 2011
**Trans−AT KS**	trans-AT Ketosynthase	Yadav et al. 2009
**Trp halogenase**	Tryptophan halogenase	PF04820.9

Table S5. Summary of TCDB classification system presented in this study

superfamily ID	superfamily description	family ID	family description
**TCSF01**	AAA-ATPase	1.F.1	The Synaptosomal Vesicle Fusion Pore (SVF-Pore) Family
**TCSF01**	AAA-ATPase	1.R.1	The Membrane Contact Site (MCS) Family
**TCSF01**	AAA-ATPase	3.A.9	The Chloroplast Envelope Protein Translocase (CEPT or Tic-Toc) Family
**TCSF01**	AAA-ATPase	3.A.16	The Endoplasmic Reticular Retrotranslocon (ER-RT) Family
**TCSF01**	AAA-ATPase	3.A.20	The Peroxisomal Protein Importer (PPI) Family
**TCSF01**	AAA-ATPase	3.A.23	The Type VI Symbiosis/Virulence Secretory System (T6SS) Family
**TCSF01**	AAA-ATPase	3.A.24	The Type VII or ESX Protein Secretion System (T7SS) Family
**TCSF01**	AAA-ATPase	3.A.25	The Symbiont-specific ERAD-like Machinery (SELMA) Family
**TCSF01**	AAA-ATPase	3.A.28	The AAA-ATPase, Bcs1 (Bcs1) Family
**TCSF01**	AAA-ATPase	3.A.29	The Mitochondrial Inner Membrane i-AAA Protease Complex (MIMP) Familly
**TCSF01**	AAA-ATPase	3.A.31	The Endosomal Sorting Complexes Required for Transport III (ESCRT-III) Family
**TCSF02**	ABC1, ABC2, ABC3	2.A.87	The Prokaryotic Riboflavin Transporter (P-RFT) Family
**TCSF02**	ABC1, ABC2, ABC3	2.A.88	The Vitamin Uptake Transporter (VUT) Family
**TCSF02**	ABC1, ABC2, ABC3	3.A.1	The ATP-binding Cassette (ABC) Superfamily
**TCSF03**	A-OMP	1.B.89	The Gordonia Outer Membrane Porin (GjpA) Family
**TCSF03**	A-OMP	1.B.94	The Pro-Pro-Glu Outer Membrane Porin (PPE) Family
**TCSF03**	A-OMP	9.B.96	The PE-PGRS Protein (PE-PGRS) Family
**TCSF04**	A/GCyc	8.A.59	The SLC and TCST-Associated Component (STAC-A) Family
**TCSF04**	A/GCyc	8.A.85	The Guanylate Cyclase (GC) Family
**TCSF04**	A/GCyc	9.B.33	The Sensor Histidine Kinase (SHK) Family
**TCSF05**	Aerolysin	1.C.3	The α-Hemolysin Channel-forming Toxin (αHL) Family
**TCSF05**	Aerolysin	1.C.4	The Aerolysin Channel-forming Toxin (Aerolysin) Family
**TCSF05**	Aerolysin	1.C.5	The Channel-forming ε-toxin (ε-toxin) Family
**TCSF05**	Aerolysin	1.C.13	The Channel-forming Leukocidin Cytotoxin (Ctx) Family
**TCSF05**	Aerolysin	1.C.14	The Cytohemolysin (CHL) Family
**TCSF05**	Aerolysin	1.C.43	The Earthworm Lysenin Toxin (Lysenin) Family
**TCSF05**	Aerolysin	1.C.74	The Snake Cytotoxin (SCT) Family
**TCSF05**	Aerolysin	1.C.78	The Crystal Protein (Cry) Family
**TCSF05**	Aerolysin	8.A.31	The Ly-6 Neurotoxin-like Protein1 Precursor (Lynx1) Family
**TCSF05**	Aerolysin	8.B.23	The Mambalgin (Mambalgin) Family
**TCSF06**	Ank	1.I.1	The Nuclear Pore Complex (NPC) Family
**TCSF06**	Ank	1.C.63	The α-Latrotoxin (Latrotoxin) Family
**TCSF06**	Ank	1.A.105	The Mixed Lineage Kinase Domain-like (MLKL) Family

**TCSF06**	Ank	1.C.104	The Heterokaryon Incompatibility Prion/Amyloid Protein (HET-s) Family
**TCSF06**	Ank	3.A.5	The General Secretory Pathway (Sec) Family
**TCSF06**	Ank	8.A.23	The Basigin (Basigin) Family
**TCSF06**	Ank	8.A.28	The Ankyrin (Ankyrin) Family
**TCSF06**	Ank	8.A.35	The Mycobacterial Membrane Protein Small (MmpS) Family
**TCSF06**	Ank	9.A.43	The Cadmium Tolerance Efflux Pump (CTEP) Family
**TCSF06**	Ank	9.B.87	The Selenoprotein P Receptor (SelP-Receptor) Family
**TCSF07**	Anoctamin	1.A.17	The Calcium-dependent Chloride Channel (Ca-ClC) Family
**TCSF08**	APC	2.A.3	The Amino Acid-Polyamine-Organocation (APC) Family
**TCSF08**	APC	2.A.15	The Betaine/Carnitine/Choline Transporter (BCCT) Family
**TCSF08**	APC	2.A.18	The Amino Acid/Auxin Permease (AAAP) Family
**TCSF08**	APC	2.A.21	The Solute:Sodium Symporter (SSS) Family
**TCSF08**	APC	2.A.22	The Neurotransmitter:Sodium Symporter (NSS) Family
**TCSF08**	APC	2.A.25	The Alanine or Glycine:Cation Symporter (AGCS) Family
**TCSF08**	APC	2.A.26	The Branched Chain Amino Acid:Cation Symporter (LIVCS) Family
**TCSF08**	APC	2.A.30	The Cation-Chloride Cotransporter (CCC) Family
**TCSF08**	APC	2.A.31	The Anion Exchanger (AE) Family
**TCSF08**	APC	2.A.39	The Nucleobase:Cation Symporter-1 (NCS1) Family
**TCSF08**	APC	2.A.40	The Nucleobase/Ascorbate Transporter (NAT) or Nucleobase:Cation Symporter-2 (NCS2) Family
**TCSF08**	APC	2.A.42	The Hydroxy/Aromatic Amino Acid Permease (HAAAP) Family
**TCSF08**	APC	2.A.46	The Benzoate:H+ Symporter (BenE) Family
**TCSF08**	APC	2.A.53	The Sulfate Permease (SulP) Family
**TCSF08**	APC	2.A.55	The Metal Ion (Mn2+-iron) Transporter (Nramp) Family
**TCSF08**	APC	2.A.72	The K+ Uptake Permease (KUP) Family
**TCSF08**	APC	2.A.114	The Putative Peptide Transporter Carbon Starvation CstA (CstA) Family
**TCSF08**	APC	2.A.120	The Putative Amino Acid Permease (PAAP) Family
**TCSF09**	ArsA	3.A.4	The Arsenite-Antimonite (ArsAB) Efflux Family
**TCSF10**	BB	1.C.22	The Lactococcin A (Lactococcin A) Family
**TCSF10**	BB	1.C.24	The Pediocin (Pediocin) Family
**TCSF10**	BB	1.C.26	The Lactacin X (Lactacin X) Family
**TCSF10**	BB	1.C.27	The Divergicin A (Divergicin A) Family
**TCSF10**	BB	1.C.29	The Plantaricin EF (Plantaricin EF) Family
**TCSF10**	BB	1.C.30	The Plantaricin JK (Plantaricin JK) Family
**TCSF10**	BB	1.C.31	The Channel-forming Colicin V (Colicin V) Family
**TCSF10**	BB	1.C.53	The Lactocyclicin Q (Lactocyclicin Q) Family
**TCSF10**	BB	1.C.102	The Cerein (Cerein) Family
**TCSF11**	BART	2.A.10	The 2-Keto-3-Deoxygluconate Transporter (KdgT) Family

**TCSF11**	BART	2.A.28	The Bile Acid:Na+ Symporter (BASS) Family
**TCSF11**	BART	2.A.59	The Arsenical Resistance-3 (ACR3) Family
**TCSF11**	BART	2.A.69	The Auxin Efflux Carrier (AEC) Family
**TCSF11**	BART	9.B.34	The Kinase/Phosphatase/Cyclic-GMP Synthase/Cyclic di-GMP Hydrolase (KPSH) Family
**TCSF12**	Bcl-2	1.A.21	The Bcl-2 (Bcl-2) Family
**TCSF12**	Bcl-2	8.A.69	The Pro-apoptotic Bcl-2-Family Protein Bim (Bim) Family
**TCSF13**	CAAX	9.B.1	The Integral Membrane CAAX Protease (CAAX Protease) Family
**TCSF13**	CAAX	9.B.2	The Integral Membrane CAAX Protease-2 (CAAX Protease2) Family
**TCSF13**	CAAX	9.B.217	The Transmembrane PrsW Protease (PrsW) Family
**TCSF13**	CAAX	9.B.218	The DUF2324 Family of Putative Integral Membrane Metaloproteases (IMMP) Family
**TCSF13**	CAAX	9.B.220	The CAAX Protease Self-Immunity (CAAX-PSI) Family
**TCSF13**	CAAX	9.B.252	The Peptidase from Unculturable Bacteria-6 (PUB6) Family
**TCSF14**	CaCa	5.B.1	The Phagocyte (gp91phox) NADPH Oxidase Family
**TCSF14**	CaCa	8.A.82	The Calmodulin Calcium Binding Protein (Calmodulin) Family
**TCSF15**	CDF	1.A.52	The Ca2+ Release-activated Ca2+ (CRAC) Channel (CRAC-C) Family
**TCSF15**	CDF	2.A.4	The Cation Diffusion Facilitator (CDF) Family
**TCSF15**	CDF	2.A.19	The Ca2+:Cation Antiporter (CaCA) Family
**TCSF15**	CDF	2.A.103	The Bacterial Murein Precursor Exporter (MPE) Family
**TCSF16**	Cecropin	1.C.17	The Cecropin (Cecropin) Family
**TCSF16**	Cecropin	1.C.18	The Melittin (Melittin) Family
**TCSF16**	Cecropin	1.C.32	The Amphipathic Peptide Mastoparan (Mastoparan) Family
**TCSF16**	Cecropin	1.C.51	The Pilosulin (Pilosulin) Family
**TCSF16**	Cecropin	1.C.52	The Dermaseptin (Dermaseptin) Family
**TCSF16**	Cecropin	1.C.62	The Pseudopleuronectes americanus (flounder) Pleurocidin (Pleurocidin) Family
**TCSF16**	Cecropin	1.C.76	The Pore-forming Maculatin Peptide (Maculatin) Family
**TCSF16**	Cecropin	1.C.124	The Antimicrobial Pore-forming Pandinin (Pin) Family
**TCSF17**	CBB	1.C.28	The Bacteriocin AS-48 Cyclic Polypeptide (Bacteriocin AS-48) Family
**TCSF17**	CBB	1.C.83	The Gassericin (Gassericin) Family
**TCSF17**	CBB	1.C.84	The Subtilosin (Subtilosin) Family
**TCSF17**	CBB	1.C.90	The Carnocyclin A (Carnocyclin) Family
**TCSF18**	CNNM/HlyC	1.A.112	The Cyclin M Mg2+ Exporter (CNNM) Family
**TCSF18**	CNNM/HlyC	1.C.126	The HlyC HlyC) Family of Haemolysins
**TCSF19**	Conotoxin	8.B.10	The Psalmotoxin-1 (PcTx1) Family
**TCSF19**	Conotoxin	8.B.20	The Australian Scorpion Toxin (Liotoxin) Family
**TCSF19**	Conotoxin	8.B.22	The P-Conotoxin Cystine Knot (P-CCK) Family
**TCSF19**	Conotoxin	8.B.28	The Mu-Conotoxin (Mu-Conotoxin) Family

**TCSF19**	Conotoxin	8.B.32	The Nicotinic AcetylCholine Receptor-targeting Alpha-Conotoxin (A-Conotoxin) Family
**TCSF19**	Conotoxin	8.B.34	The Sigma-Conotoxin (Sigma-Conotoxin) Family
**TCSF19**	Conotoxin	8.B.36	The Contulakin Lt (Contulakin Lt) Family
**TCSF19**	Conotoxin	8.B.40	The Conotoxin Con-ikot-ikot/Conopressin/Conophysin/Conodipine (CCCC) Family
**TCSF20**	CuR	9.A.55	The TMEM205 (TMEM205) Family
**TCSF20**	CuR	9.B.62	The Copper Resistance (CopD) Family
**TCSF21**	CPA	2.A.27	The Glutamate:Na+ Symporter (ESS) Family
**TCSF21**	CPA	2.A.36	The Monovalent Cation:Proton Antiporter-1 (CPA1) Family
**TCSF21**	CPA	2.A.37	The Monovalent Cation:Proton Antiporter-2 (CPA2) Family
**TCSF21**	CPA	2.A.70	The Malonate:Na+ Symporter (MSS) Family
**TCSF21**	CPA	2.A.81	The Aspartate:Alanine Exchanger (AAEx) Family
**TCSF21**	CPA	2.A.98	The Putative Sulfate Exporter (PSE) Family
**TCSF21**	CPA	3.B.1	The Na+-transporting Carboxylic Acid Decarboxylase (NaT-DC) Family
**TCSF22**	Defensin	1.C.19	The Defensin (Defensin) Family
**TCSF22**	Defensin	1.C.45	The Plant Defensin (Plant Defensin) Family
**TCSF22**	Defensin	1.C.47	The Insect/Fungal Defensin (Insect/Fungal Defensin) Family
**TCSF22**	Defensin	1.C.85	The Pore-Forming β-Defensin (β-Defensin) Family
**TCSF22**	Defensin	8.B.1	The Long (4C-C) Scorpion Toxin (L-ST) Superfamily
**TCSF22**	Defensin	8.B.2	The Short Scorpion Toxin (S-ST) Superfamily
**TCSF22**	Defensin	8.B.7	The Cl- Channel Peptide Inhibitor (GaTx1) Family
**TCSF22**	Defensin	8.B.8	The α-KTx15 scorpion toxin (α-KTx15) Family
**TCSF23**	DMT	2.A.7	The Drug/Metabolite Transporter (DMT) Superfamily
**TCSF24**	ENaC/P2X	1.A.6	The Epithelial Na+ Channel (ENaC) Family
**TCSF24**	ENaC/P2X	1.A.7	The ATP-gated P2X Receptor Cation Channel (P2X Receptor) Family
**TCSF26**	GTT	4.D.1	The Putative Vectorial Glycosyl Polymerization (VGP) Family
**TCSF26**	GTT	4.D.2	The Glycosyl Transferase 2 (GT2) Family
**TCSF26**	GTT	4.D.3	The Glycan Glucosyl Transferase (OpgH) Family
**TCSF27**	GAT	9.B.97	The Acyltransferase-3/Putative Acetyl-CoA Transporter (ATAT) Family
**TCSF27**	GAT	9.B.142	The Integral membrane Glycosyltransferase family 39 (GT39) Family
**TCSF27**	GAT	9.B.239	The Uncharacterized Bacterial 10 - 13 TMS Protein (UBP2) Family
**TCSF30**	GET	3.A.19	The TMS Recognition/Insertion Complex (TRC) Family
**TCSF30**	GET	3.A.21	The C-terminal Tail-Anchored Membrane Protein Biogenesis/ Insertion Complex (TAMP-B) Family
**TCSF31**	Holin I	1.E.11	The φ11 Holin (φ11 Holin) Family
**TCSF32**	Holin II	1.E.1	The P21 Holin S (P21 Holin) Family
**TCSF32**	Holin II	1.E.6	The T7 Holin (T7 Holin) Family
**TCSF32**	Holin II	1.E.7	The HP1 Holin (HP1 Holin) Family

**TCSF32**	Holin II	1.E.25	The Pseudomonas phage F116 Holin (F116 Holin) Family
**TCSF32**	Holin II	1.E.50	The Beta-Proteobacterial Holin (BP-Hol) Family
**TCSF33**	Holin III	1.E.2	The λ Holin S (λ Holin) Family
**TCSF33**	Holin III	1.E.3	The P2 Holin (P2 Holin) Family
**TCSF33**	Holin III	1.E.4	The LydA Holin (LydA Holin) Family
**TCSF33**	Holin III	1.E.5	The PRD1 Phage P35 Holin (P35 Holin) Family
**TCSF33**	Holin III	1.E.20	The Pseudomonas aeruginosa Hol Holin (Hol Holin) Family
**TCSF33**	Holin III	1.E.34	The Putative Actinobacterial Holin-X (Hol-X) Family
**TCSF33**	Holin III	1.E.41	The Deinococcus/Thermus Holin (D/T-Hol) Family
**TCSF34**	Holin IV	1.E.10	The Bacillus subtilis φ29 Holin (φ29 Holin) Family
**TCSF34**	Holin IV	1.E.16	The Cph1 Holin (Cph1 Holin) Family
**TCSF34**	Holin IV	1.E.19	The Clostridium difficile TcdE Holin (TcdE Holin) Family
**TCSF34**	Holin IV	1.E.40	The Mycobacterial 4 TMS Phage Holin (MP4 Holin) Family
**TCSF34**	Holin IV	1.E.43	The Putative Transglycosylase-associated Holin (T-A Hol) Family
**TCSF35**	Holin V	1.E.21	The Listeria Phage A118 Holin (Hol118) Family
**TCSF35**	Holin V	1.E.29	The Holin Hol44 (Hol44) Family
**TCSF36**	Holin VI	1.E.12	The φAdh Holin (φAdh Holin) Family
**TCSF36**	Holin VI	1.E.26	The Holin LLH (Holin LLH) Family
**TCSF37**	Holin VII	1.E.36	The Mycobacterial 2 TMS Phage Holin (M2 Hol) Family
**TCSF38**	Huwentoxin	8.B.3	The Huwentoxin-1 (Huwentoxin-1) Family
**TCSF38**	Huwentoxin	8.B.4	The Conotoxin T (Conotoxin T) Family
**TCSF38**	Huwentoxin	8.B.5	The Na+/K+/Ca2+ Channel Targeting Tarantula Huwentoxin (THT) Family
**TCSF38**	Huwentoxin	8.B.6	The Ca2+ Channel-targeting Spider Toxin (CST) Family
**TCSF38**	Huwentoxin	8.B.12	The Spider Toxin (STx2) Family
**TCSF38**	Huwentoxin	8.B.16	The Maurocalcine (MaCa) Family
**TCSF38**	Huwentoxin	8.B.19	The Sea Anemone K+ Channel Blocker Toxin, BcsTx3 (BcsTx3) Family
**TCSF38**	Huwentoxin	8.B.21	The Spider Insecticidal Neurotoxin Cyrtautoxin (Cyrautoxin) Family
**TCSF39**	Hydrolase	4.C.3	The Acyl-CoA Thioesterase (ACoA-T) Family
**TCSF39**	Hydrolase	8.A.51	The Dipeptidyl-aminopeptidase-like Protein 6 beta subunit of Kv4 channels (DPP6) Family
**TCSF39**	Hydrolase	9.B.371	The PaaI Thioesterase (PTE) Family
**TCSF40**	M2	1.A.19	The Type A Influenza Virus Matrix-2 Channel (M2-C) Family
**TCSF40**	M2	1.A.58	The Type B Influenza Virus Matrix Protein 2 (BM2-C) Family
**TCSF41**	ISP	3.D.1	The H+ or Na+-translocating NADH Dehydrogenase (NDH) Family
**TCSF41**	ISP	3.D.6	The Ion (H+ or Na+)-translocating NADH:Ferredoxin Oxidoreductase (NFO or RNF) Family
**TCSF41**	ISP	3.D.7	The H2:Heterodisulfide Oxidoreductase (HHO) Family
**TCSF41**	ISP	3.D.8	The Na+- or H+-Pumping Formyl Methanofuran Dehydrogenase (FMF-DH) Family
**TCSF41**	ISP	3.D.9	The H+-translocating F420H2 Dehydrogenase (F420H2DH) Family

**TCSF41**	ISP	3.D.10	The Prokaryotic Succinate Dehydrogenase (SDH) Family
**TCSF41**	ISP	3.D.11	The Periplasmic Nitrate Reductase Complex (NAP) Complex Family
**TCSF41**	ISP	5.A.3	The Prokaryotic Molybdopterin-containing Oxidoreductase (PMO) Family
**TCSF41**	ISP	5.B.4	The Plant Photosystem I Supercomplex (PSI) Family
**TCSF41**	ISP	5.B.11	The One Electron Transmembrane Transfer Complex (TmcABCD) Family
**TCSF41**	ISP	5.B.12	The One Electron Transmembrane Transfer Complex (DsrMKJOP) Family
**TCSF41**	ISP	5.B.13	The One Electron Transmembrane Transfer Complex (HmcABCDEF) Family
**TCSF42**	IT	2.A.8	The Gluconate:H+ Symporter (GntP) Family
**TCSF42**	IT	2.A.11	The Citrate-Mg2+:H+ (CitM) Citrate-Ca2+:H+ (CitH) Symporter (CitMHS) Family
**TCSF42**	IT	2.A.13	The C4-Dicarboxylate Uptake (Dcu) Family
**TCSF42**	IT	2.A.14	The Lactate Permease (LctP) Family
**TCSF42**	IT	2.A.34	The NhaB Na+:H+ Antiporter (NhaB) Family
**TCSF42**	IT	2.A.35	The NhaC Na+:H+ Antiporter (NhaC) Family
**TCSF42**	IT	2.A.45	The Arsenite-Antimonite (ArsB) Efflux Family
**TCSF42**	IT	2.A.47	The Divalent Anion:Na+ Symporter (DASS) Family
**TCSF42**	IT	2.A.56	The Tripartite ATP-independent Periplasmic Transporter (TRAP-T) Family
**TCSF42**	IT	2.A.61	The C4-dicarboxylate Uptake C (DcuC) Family
**TCSF42**	IT	2.A.62	The NhaD Na+:H+ Antiporter (NhaD) Family
**TCSF42**	IT	2.A.68	The p-Aminobenzoyl-glutamate Transporter (AbgT) Family
**TCSF42**	IT	2.A.94	The Phosphate Permease (Pho1) Family
**TCSF42**	IT	2.A.101	The Malonate Uptake (MatC) Family (Formerly UIT1)
**TCSF42**	IT	2.A.111	The Na+/H+ Antiporter-E (NhaE) Family
**TCSF42**	IT	2.A.118	The Basic Amino Acid Antiporter (ArcD) Family
**TCSF43**	La-Ba	1.C.21	The Lacticin 481 (Lacticin 481) Family
**TCSF43**	La-Ba	1.C.60	The Two-component Enterococcus faecalis Cytolysin (EFC) Family
**TCSF44**	LRRD	1.A.87	The Mechanosensitive Calcium Channel (MCA) Family
**TCSF44**	LRRD	8.A.43	The NEAT-domain containing methaemoglobin heme sequestration (N-MHS) Family
**TCSF44**	LRRD	9.A.5	The Putative Arginine Transporter (ArgW) Family
**TCSF45**	LysE	2.A.75	The L-Lysine Exporter (LysE) Family
**TCSF45**	LysE	2.A.76	The Resistance to Homoserine/Threonine (RhtB) Family
**TCSF45**	LysE	2.A.77	The Cadmium Resistance (CadD) Family
**TCSF45**	LysE	2.A.95	The 6 TMS Neutral Amino Acid Transporter (NAAT) Family
**TCSF45**	LysE	2.A.106	The Ca2+:H+ Antiporter-2 (CaCA2) Family
**TCSF45**	LysE	2.A.107	The MntP Mn2+ exporter (MntP) Family
**TCSF45**	LysE	2.A.108	The Iron/Lead Transporter (ILT) Family
**TCSF45**	LysE	2.A.113	The Nickel/cobalt Transporter (NicO) Family

**TCSF45**	LysE	2.A.116	The Peptidoglycolipid Addressing Protein (GAP) Family
**TCSF45**	LysE	2.A.131	The Aminobenzyl Carbon-arsenic Defining Exporter (ABCDE) Family
**TCSF45**	LysE	5.A.1	The Disulfide Bond Oxidoreductase D (DsbD) Family
**TCSF46**	MACPF	1.C.12	The Thiol-activated Cholesterol-dependent Cytolysin (CDC) Family
**TCSF46**	MACPF	1.C.39	The Membrane Attack Complex/Perforin (MACPF) Family
**TCSF46**	MACPF	1.C.97	The Pleurotolysin Pore-forming (Pleurotolysin) Family
**TCSF47**	MFS	2.A.1	The Major Facilitator Superfamily (MFS)
**TCSF47**	MFS	2.A.2	The Glycoside-Pentoside-Hexuronide (GPH):Cation Symporter Family
**TCSF47**	MFS	2.A.12	The ATP:ADP Antiporter (AAA) Family
**TCSF47**	MFS	2.A.17	The Proton-dependent Oligopeptide Transporter (POT/PTR) Family
**TCSF47**	MFS	2.A.48	The Reduced Folate Carrier (RFC) Family
**TCSF47**	MFS	2.A.57	The Equilibrative Nucleoside Transporter (ENT) Family
**TCSF47**	MFS	2.A.60	The Organo Anion Transporter (OAT) Family
**TCSF47**	MFS	2.A.71	The Folate-Biopterin Transporter (FBT) Family
**TCSF47**	MFS	2.A.85	The Aromatic Acid Exporter (ArAE) Family
**TCSF47**	MFS	2.A.100	The Ferroportin (Fpn) Family
**TCSF47**	MFS	2.A.125	The Eukaryotic Riboflavin Transporter (E-RFT) Family
**TCSF47**	MFS	4.H.1	The Lysyl phosphatidylglycerol Synthase/Flippase (MprF) Family
**TCSF47**	MFS	5.B.2	The Eukaryotic Cytochrome b561 (Cytb561) Family
**TCSF47**	MFS	9.B.57	The Conidiation and Conidial Germination Protein (CCGP) Family
**TCSF47**	MFS	9.B.111	The 6 TMS Lysyl tRNA Synthetase (LysS) Family
**TCSF47**	MFS	9.B.143	The 6 TMS DUF1275/Pf06912 (DUF1275) Family
**TCSF48**	MIP	1.A.8	The Major Intrinsic Protein (MIP) Family
**TCSF48**	MIP	1.A.16	The Formate-Nitrite Transporter (FNT) Family
**TCSF49**	MFP	1.G.1	The Viral Pore-forming Membrane Fusion Protein-1 (VMFP1) Family
**TCSF49**	MFP	1.G.14	The Influenza Virus Hemagglutinin/Fusion Pore-forming Protein (Influenza-H/FPP) Family
**TCSF50**	Mer	1.A.72	The Mer Superfamily
**TCSF51**	MC	2.A.29	The Mitochondrial Carrier (MC) Family
**TCSF52**	MOP	2.A.66	The Multidrug/Oligosaccharidyl-lipid/Polysaccharide (MOP) Flippase Superfamily
**TCSF53**	Mrp	2.A.63	The Monovalent Cation (K+ or Na+):Proton Antiporter-3 (CPA3) Family
**TCSF54**	OAPol	9.B.67	The O-antigen Polymerase (OAP) Family
**TCSF54**	OAPol	9.B.128	The O-antigen Polymerase, WzyE (WzyE) Family
**TCSF55**	OMPP-I	1.B.1	The General Bacterial Porin (GBP) Family
**TCSF55**	OMPP-I	1.B.2	The Chlamydial Porin (CP) Family
**TCSF55**	OMPP-I	1.B.3	The Sugar Porin (SP) Family
**TCSF55**	OMPP-I	1.B.4	The Brucella-Rhizobium Porin (BRP) Family

**TCSF55**	OMPP-I	1.B.5	The Pseudomonas OprP Porin (POP) Family
**TCSF55**	OMPP-I	1.B.6	The OmpA-OmpF Porin (OOP) Family
**TCSF55**	OMPP-I	1.B.7	The Rhodobacter PorCa Porin (RPP) Family
**TCSF55**	OMPP-I	1.B.8	The Mitochondrial and Plastid Porin (MPP) Family
**TCSF55**	OMPP-I	1.B.9	The FadL Outer Membrane Protein (FadL) Family
**TCSF55**	OMPP-I	1.B.10	The Nucleoside-specific Channel-forming Outer Membrane Porin (Tsx) Family
**TCSF55**	OMPP-I	1.B.11	The Outer Membrane Fimbrial Usher Porin (FUP) Family
**TCSF55**	OMPP-I	1.B.12	The Autotransporter-1 (AT-1) Family
**TCSF55**	OMPP-I	1.B.13	The Alginate Export Porin (AEP) Family
**TCSF55**	OMPP-I	1.B.14	The Outer Membrane Receptor (OMR) Family
**TCSF55**	OMPP-I	1.B.15	The Raffinose Porin (RafY) Family
**TCSF55**	OMPP-I	1.B.16	The Short Chain Amide and Urea Porin (SAP) Family
**TCSF55**	OMPP-I	1.B.17	The Outer Membrane Factor (OMF) Family
**TCSF55**	OMPP-I	1.B.18	The Outer Membrane Auxiliary (OMA) Protein Family
**TCSF55**	OMPP-I	1.B.19	The Glucose-selective OprB Porin (OprB) Family
**TCSF55**	OMPP-I	1.B.20	The Two-Partner Secretion (TPS) Family
**TCSF55**	OMPP-I	1.B.21	The OmpG Porin (OmpG) Family
**TCSF55**	OMPP-I	1.B.22	The Outer Bacterial Membrane Secretin (Secretin) Family
**TCSF55**	OMPP-I	1.B.23	The Cyanobacterial Porin (CBP) Family
**TCSF55**	OMPP-I	1.B.25	The Outer Membrane Porin (Opr) Family
**TCSF55**	OMPP-I	1.B.26	The Cyclodextrin Porin (CDP) Family
**TCSF55**	OMPP-I	1.B.31	The Campylobacter jejuni Major Outer Membrane Porin (MomP) Family
**TCSF55**	OMPP-I	1.B.32	The Fusobacterial Outer Membrane Porin (FomP) Family
**TCSF55**	OMPP-I	1.B.33	The Outer Membrane Protein Insertion Porin (Bam Complex) (OmpIP) Family
**TCSF55**	OMPP-I	1.B.35	The Oligogalacturonate-specific Porin (KdgM) Family
**TCSF55**	OMPP-I	1.B.39	The Bacterial Porin, OmpW (OmpW) Family
**TCSF55**	OMPP-I	1.B.42	The Outer Membrane Lipopolysaccharide Export Porin (LPS-EP) Family
**TCSF55**	OMPP-I	1.B.43	The Coxiella Porin P1 (CPP1) Family
**TCSF55**	OMPP-I	1.B.44	The Probable Protein Translocating Porphyromonas gingivalis Porin (PorT) Family
**TCSF55**	OMPP-I	1.B.49	The Anaplasma P44 (A-P44) Porin Family
**TCSF55**	OMPP-I	1.B.54	The Intimin/Invasin (Int/Inv) or Autotransporter-3 (AT-3) Family
**TCSF55**	OMPP-I	1.B.55	The Poly Acetyl Glucosamine Porin (PgaA) Family
**TCSF55**	OMPP-I	1.B.57	The Legionella Major-Outer Membrane Protein (LM-OMP) Family
**TCSF55**	OMPP-I	1.B.60	The Omp50 Porin (Omp50 Porin) Family
**TCSF55**	OMPP-I	1.B.61	The Delta-Proteobacterial Porin (Delta-Porin) Family
**TCSF55**	OMPP-I	1.B.62	The Putative Bacterial Porin (PBP) Family
**TCSF55**	OMPP-I	1.B.66	The Putative Beta-Barrel Porin-2 (BBP2) Family

**TCSF55**	OMPP-I	1.B.67	The Putative Beta Barrel Porin-4 (BBP4) Family
**TCSF55**	OMPP-I	1.B.68	The Putative Beta Barrel Porin-5 (BBP5) Superfamily
**TCSF55**	OMPP-I	1.B.70	The Outer Membrane Channel (OMC) Family
**TCSF55**	OMPP-I	1.B.71	The Proteobacterial/Verrucomicrobial Porin (PVP) Family
**TCSF55**	OMPP-I	1.B.72	The Protochlamydial Outer Membrane Porin (PomS/T) Family
**TCSF55**	OMPP-I	1.B.73	The Capsule Biogenesis/Assembly (CBA) Family
**TCSF55**	OMPP-I	1.B.78	The DUF3374 Electron Transport-associated Porin (ETPorin) Family
**TCSF55**	OMPP-I	1.B.80	The Putative Trans-Outer Membrane Electron Flow Porin (TOM-EF) Family
**TCSF55**	OMPP-I	1.B.81	The DUF2490 Putative Beta Barrel Porin (DUF2490) Family
**TCSF55**	OMPP-I	1.B.92	The Proteobacterial Outer Membrane Porin, NilB (NilB) Family
**TCSF55**	OMPP-I	1.B.95	The Outer Membrane Protein YaiO (YaiO) Family
**TCSF55**	OMPP-I	9.B.50	The Outer Membrane Beta-barrel Endoprotease, Omptin (Omptin) Family
**TCSF55**	OMPP-I	9.B.153	The Putative Beta-Barrel Porin/Alpha Amylase or Phenol_MetA-deg (BBP/AA) Family
**TCSF55**	OMPP-I	9.B.170	The DUF3187 Putative Porin (DUF3187) Family
**TCSF55**	OMPP-I	9.B.186	The Putative Lipoprotein Supressor of a ts bamD mutant, YiaD (YiaD) Family
**TCSF56**	OMPP-II	1.B.24	The Mycobacterial Porin (MBP) Family
**TCSF56**	OMPP-II	1.B.58	The Nocardial Hetero-oligomeric Cell Wall Channel (NfpA/B) Family
**TCSF57**	OMPP-III	1.B.28	The Plastid Outer Envelope Porin of 24 kDa (OEP24) Family
**TCSF57**	OMPP-III	1.B.47	The Plastid Outer Envelope Porin of 37 kDa (OEP37) Family
**TCSF58**	OMPP-IV	1.B.30	The Plastid Outer Envelope Porin of 16 kDa (OEP16) Family
**TCSF58**	OMPP-IV	1.B.69	The Peroxysomal Membrane Porin 4 (PxMP4) Family
**TCSF58**	OMPP-IV	3.A.8	The Mitochondrial Protein Translocase (MPT) Family
**TCSF59**	OMPP-V	1.B.34	The Corynebacterial Porin A (PorA) Family
**TCSF59**	OMPP-V	1.B.59	The Outer Membrane Porin, PorH (PorH) Family
**TCSF60**	P-ATPase	3.A.3	The P-type ATPase (P-ATPase) Superfamily
**TCSF61**	Pex	1.A.101	The Peroxisomal Pore-forming Pex11 (Pex11) Family
**TCSF62**	PPP	1.W.1	The Phage Portal Protein 1 (PPP1) Family
**TCSF62**	PPP	1.W.2	The Phage Portal Protein 2 (PPP2) Family
**TCSF62**	PPP	1.W.3	The Phage Portal Protein 3 (PPP3) Family
**TCSF62**	PPP	1.W.5	The (Lambda) Phage Portal Protein 5 (PPP5) Family
**TCSF62**	PPP	1.W.7	The (Bacillus Phage SPP1) Portal Protein 7 (PPP7) Family
**TCSF62**	PPP	1.W.8	The (Enterobacterial phage T4) Portal Protein 8 (PPP8) Family
**TCSF62**	PPP	1.W.9	The (Eschericha coli Mu) Phage Portal Portein 9 (PPP9) Family
**TCSF62**	PPP	1.W.10	The (Enterobacterial Phage T7) Portal Protein 10 (PPP10) Family
**TCSF63**	PSV	1.A.27	The Phospholemman (PLM) Family
**TCSF63**	PSV	1.A.95	The Ephemerovirus Viroporin (EVVP) Family
**TCSF63**	PSV	1.A.100	The Rhabdoviridae Putative Viroporin, U5 (RV-U5) Family

**TCSF63**	PSV	1.A.113	The Small Integral Membrane Protein (SIMP) Family
**TCSF64**	PL-TP	2.A.127	The Enterobacterial Cardiolipin Transporter (CLT) Family
**TCSF64**	PL-TP	9.B.105	The Lead Resistance Fusion Protein (PbrBC) Family
**TCSF65**	PoPo	4.E.1	The Vacuolar (Acidocalcisome) Polyphosphate Polymerase (V-PPP) Family
**TCSF65**	PoPo	9.B.51	The Uncharacterized DUF202/YidH (YidH) Family
**TCSF66**	PfCTx	1.C.41	The Tripartite Haemolysin BL (HBL) Family
**TCSF67**	PK	8.A.104	The 5'-AMP-activated protein kinase (AMPK) Family
**TCSF67**	PK	9.A.15	The Autophagy-related Phagophore-formation Transporter (APT) Family
**TCSF67**	PK	9.B.106	The Pock Size-determining Protein (PSDP) Family
**TCSF68**	PTS-AG	4.A.5	The PTS Galactitol (Gat) Family
**TCSF68**	PTS-AG	4.A.7	The PTS L-Ascorbate (L-Asc) Family
**TCSF69**	PTS-GFL	4.A.1	The PTS Glucose-Glucoside (Glc) Family
**TCSF69**	PTS-GFL	4.A.2	The PTS Fructose-Mannitol (Fru) Family
**TCSF69**	PTS-GFL	4.A.3	The PTS Lactose-N,N'-Diacetylchitobiose-β-glucoside (Lac) Family
**TCSF69**	PTS-GFL	4.A.4	The PTS Glucitol (Gut) Family
**TCSF70**	RND	2.A.6	The Resistance-Nodulation-Cell Division (RND) Superfamily
**TCSF71**	Retromer	9.A.3	The Sorting Nexin27 (SNX27)-Retromer Assembly Apparatus
**TCSF71**	Retromer	9.A.63	The Retromer-dependent Vacuolar Protein Sorting (R-VPS) Family
**TCSF72**	RTX-toxin	1.C.11	The Pore-forming RTX Toxin (RTX-toxin) Family
**TCSF72**	RTX-toxin	1.C.56	The Pseudomonas syringae HrpZ Target Host Cell Membrane Cation Channel (HrpZ) Family
**TCSF72**	RTX-toxin	1.C.57	The Clostridial Cytotoxin (CCT) Family
**TCSF72**	RTX-toxin	1.C.105	The Bacillus thuringiensis Vegetative Insecticidal Protein-3 (Vip3) Family
**TCSF73**	SynapD	8.A.30	The Nedd4-Family Interacting Protein-2 (Nedd4) Family
**TCSF73**	SynapD	8.A.78	The Insulin Secretion-regulating Lipid Transporter TMEM24 (TMEM24) Family
**TCSF73**	SynapD	9.A.48	The Unconventional Protein Secretion (UPS) System
**TCSF73**	SynapD	9.A.57	The Extended-Synaptotagmin (E-Syt) Family
**TCSF74**	4JC	1.H.1	The Claudin Tight Junction (Claudin1) Family
**TCSF74**	4JC	1.H.2	The Invertebrate PMP22-Claudin (Claudin2) Family
**TCSF74**	4JC	1.A.24	The Gap Junction-forming Connexin (Connexin) Family
**TCSF74**	4JC	1.A.25	The Gap Junction-forming Innexin (Innexin) Family
**TCSF74**	4JC	1.A.36	The Intracellular Chloride Channel (ICC) Family
**TCSF74**	4JC	1.A.64	The Plasmolipin (Plasmolipin) Family
**TCSF74**	4JC	1.A.81	The Low Affinity Ca2+ Channel (LACC) Family
**TCSF74**	4JC	1.A.82	The LHFPL Tetraspan Protein (LTSP) Family
**TCSF74**	4JC	1.A.84	The Calcium Homeostasis Modulator Ca2+ Channel (CALHM-C) Family
**TCSF74**	4JC	8.A.16	The Ca+ Channel Auxiliary Subunit γ1-γ8 (CCAγ) Family

**TCSF74**	4JC	9.A.27	The Non-Classical Protein Exporter (NCPE) Family
**TCSF74**	4JC	9.A.46	The Clarin (CLRN) Family
**TCSF74**	4JC	9.B.41	The Occludin (Occludin) Family
**TCSF74**	4JC	9.B.130	The Tetraspan Vesicle Membrane Protein (TVP) Family
**TCSF74**	4JC	9.B.179	The MscS/DUF475 (DUF475) Family
**TCSF75**	TAPI	1.C.50	The Amyloid β-Protein Peptide (AβPP) Family
**TCSF75**	TAPI	8.B.13	The Sea Anemone Peptide Toxin Class 2 (Kalicludine) Family
**TCSF76**	TmAT	9.B.169	The Integral Membrane Protein (8 -10 TMSs) YeiB or DUF418 (YeiB) Family
**TCSF77**	TM-Cyt	5.B.3	The Geobacter Nanowire Electron Transfer (G-NET) Family
**TCSF77**	TM-Cyt	5.B.5	The Extracellular Metal Oxido-Reductase (EMOR) Family
**TCSF77**	TM-Cyt	5.B.8	The Trans-Outer Membrane Electron Transfer Porin/Cytochrome Complex (ET-PCC) Family
**TCSF78**	TOG	2.A.43	The Lysosomal Cystine Transporter (LCT) Family
**TCSF78**	TOG	2.A.52	The Ni2+-Co2+ Transporter (NiCoT) Family
**TCSF78**	TOG	2.A.82	The Organic Solute Transporter (OST) Family
**TCSF78**	TOG	2.A.102	The 4-Toluene Sulfonate Uptake Permease (TSUP) Family
**TCSF78**	TOG	2.A.104	The L-Alanine Exporter (AlaE) Family
**TCSF78**	TOG	2.A.119	The Organo-Arsenical Exporter (ArsP) Family
**TCSF78**	TOG	2.A.123	The Sweet; PQ-loop; Saliva; MtN3 (Sweet) Family
**TCSF78**	TOG	2.A.129	The Lipid-linked Sugar Translocase (LST) Family
**TCSF78**	TOG	3.E.1	The Ion-translocating Microbial Rhodopsin (MR) Family
**TCSF78**	TOG	3.E.3	The HelioRhodopsin (HelioR) Family
**TCSF78**	TOG	9.A.14	The G-protein-coupled receptor (GPCR) Family
**TCSF78**	TOG	9.B.191	The Endoplasmic Reticulum Retention Receptor (KDELR) Family
**TCSF79**	UT/RnfD/NqrB	1.A.28	The Urea Transporter (UT) Family
**TCSF79**	UT/RnfD/NqrB	3.D.5	The Na+-translocating NADH:Quinone Dehydrogenase (Na-NDH or NQR) Family
**TCSF80**	VIC	1.A.1	The Voltage-gated Ion Channel (VIC) Superfamily
**TCSF80**	VIC	1.A.2	The Inward Rectifier K+ Channel (IRK-C) Family
**TCSF80**	VIC	1.A.3	The Ryanodine-Inositol 1,4,5-triphosphate Receptor Ca2+ Channel (RIR-CaC) Family
**TCSF80**	VIC	1.A.4	The Transient Receptor Potential Ca2+ Channel (TRP-CC) Family
**TCSF80**	VIC	1.A.5	The Polycystin Cation Channel (PCC) Family
**TCSF80**	VIC	1.A.10	The Glutamate-gated Ion Channel (GIC) Family of Neurotransmitter Receptors
**TCSF80**	VIC	1.A.51	The Voltage-gated Proton Channel (VPC) Family
**TCSF80**	VIC	2.A.38	The K+ Transporter (Trk) Family
**TCSF81**	Env-FP	1.G.2	The Viral Pore-forming Membrane Fusion Protein-2 (VMFP2) Family
**TCSF81**	Env-FP	1.G.9	The Syncytin (Syncytin) Family
**TCSF81**	Env-FP	1.G.12	The Avian Leukosis Virus gp95 Fusion Protein (ALV-gp95) Family

**TCSF81**	Env-FP	1.G.17	The Bovine Leukemia Virus Envelop Glycoprotein (BLV-Env) Family
**TCSF81**	Env-FP	1.G.18	The SARS-CoV Fusion Peptide in the Spike Glycoprotein Precursor (SARS-FP) Family
**TCSF82**	YIP	9.B.29	The 4-5 TMS Putative Chaparone (4-5PC) Family
**TCSF82**	YIP	9.B.135	The Membrane Trafficking Yip (Yip) Family
